# Transcriptional Analysis of Murine Macrophages Infected with Different *Toxoplasma* Strains Identifies Novel Regulation of Host Signaling Pathways

**DOI:** 10.1371/journal.ppat.1003779

**Published:** 2013-12-19

**Authors:** Mariane B. Melo, Quynh P. Nguyen, Cynthia Cordeiro, Musa A. Hassan, Ninghan Yang, Renée McKell, Emily E. Rosowski, Lindsay Julien, Vincent Butty, Marie-Laure Dardé, Daniel Ajzenberg, Katherine Fitzgerald, Lucy H. Young, Jeroen P. J. Saeij

**Affiliations:** 1 Massachusetts Institute of Technology, Department of Biology, Cambridge, Massachusetts, United States of America; 2 Internal Medicine Department, School of Medicine, Federal University of Minas Gerais, Belo Horizonte, Minas Gerais, Brazil; 3 Retina Service, Department of Ophthalmology, Massachusetts Eye and Ear Infirmary, Harvard Medical School, Boston, Massachusetts, United States of America; 4 Centre National de Référence Toxoplasmose/Toxoplasma Biological Resource Center, Centre Hospitalier-Universitaire Dupuytren, Limoges, France; 5 Institut National de la Santé et de la Recherche Médicale, Unité Mixte de Recherche 1094, Neuroépidémiologie Tropicale, Laboratoire de Parasitologie-Mycologie, Faculté de Médecine, Université de Limoges, Limoges, France; 6 University of Massachusetts Medical School, Division of Infectious Diseases and Immunology, Worcester, Massachusetts, United States of America; National Institute of Health, United States of America

## Abstract

Most isolates of *Toxoplasma* from Europe and North America fall into one of three genetically distinct clonal lineages, the type I, II and III lineages. However, in South America these strains are rarely isolated and instead a great variety of other strains are found. *T. gondii* strains differ widely in a number of phenotypes in mice, such as virulence, persistence, oral infectivity, migratory capacity, induction of cytokine expression and modulation of host gene expression. The outcome of toxoplasmosis in patients is also variable and we hypothesize that, besides host and environmental factors, the genotype of the parasite strain plays a major role. The molecular basis for these differences in pathogenesis, especially in strains other than the clonal lineages, remains largely unexplored. Macrophages play an essential role in the early immune response against *T. gondii* and are also the cell type preferentially infected *in vivo*. To determine if non-canonical *Toxoplasma* strains have unique interactions with the host cell, we infected murine macrophages with 29 different *Toxoplasma* strains, representing global diversity, and used RNA-sequencing to determine host and parasite transcriptomes. We identified large differences between strains in the expression level of known parasite effectors and large chromosomal structural variation in some strains. We also identified novel strain-specifically regulated host pathways, including the regulation of the type I interferon response by some atypical strains. IFNβ production by infected cells was associated with parasite killing, independent of interferon gamma activation, and dependent on endosomal Toll-like receptors in macrophages and the cytoplasmic receptor retinoic acid-inducible gene 1 (RIG-I) in fibroblasts.

## Author Summary

Toxoplasmosis is caused by the protozoan parasite *Toxoplasma gondii*. The parasite is found throughout the world. When humans are infected, few have symptoms because a healthy immune system usually prevents the parasite from causing illness. Nevertheless, cases of severe disease in otherwise healthy individuals have been observed. These cases are usually a result of infection with less common atypical strains of *Toxoplasma*. Factors associated with virulence in the atypical strains are not well understood. Here, we infected host cells with 29 different strains of *Toxoplasma*, and performed high-throughput RNA sequencing of both host cells and parasites. We found significant differences in gene expression profiles between strains. Host cell transcriptional response also varied substantially depending on the infecting strain. Specifically, we found that a small group of atypical strains are able to induce production of type I interferons, which are immunomodulatory cytokines. Interferon production is a result of the elimination of internalized parasites through a novel killing mechanism. The dataset we generated is a valuable tool for identification of host cell targets of *Toxoplasma* secreted effectors and can contribute to our understanding of why certain *Toxoplasma* strains are more prone to cause severe disease in humans.

## Introduction

*Toxoplasma gondii* is a ubiquitous obligate intracellular protozoan parasite that can invade and replicate in almost all cells of a wide range of warm-blooded animals [1]. In humans it is the 2^nd^ most important foodborne pathogen in terms of annual cost of illness and quality adjusted life year loss [2]. Normally a lifelong, largely asymptomatic, infection is established but in immunocompromised individuals and in congenital infections *Toxoplasma* infection can lead to severe disease and even death. Nevertheless, not all seropositive immunosuppressed patients have reactivating toxoplasmosis [3], and not all congenital infections lead to disease [4]. There is good evidence that both the host genetic background [5]–[7] and the genotype of the infecting strain [8], [9] play a role in the severity of disease.

Despite the existence of a sexual phase in its life cycle, which only occurs in felines, few strains dominate human infections in Europe and North America. Type II strains dominate in Europe, while in North America, types 12, II and III account for the majority of strains isolated from wild-life and patients [9]–[11]. Genotypes not belonging to these lineages are predominant in South America [12]–[14]. A phylogenetic analysis of 956 strains, using single nucleotide polymorphisms (SNPs) identified in five loci, clustered these into 15 haplogroups, including type I, II and III [11], [15]–[17]. Using genome-wide SNPs, it was shown that even within these haplogroups there is often significant diversity and many strains did not fit into the 15 proposed haplogroups. Instead, many strains appear to have formed through recent recombination events [18].

The relationship between strain genotype and virulence in the mouse model is well established; type I isolates, and most South American strains, are highly virulent (LD_100_ ∼1) [19], whereas type II and III strains are less virulent, with LD_50_ of ∼10^3^ and 10^5^, respectively [20]. *Toxoplasma* invasion and modulation of its host cell is mediated by proteins secreted from three secretory organelles, called micronemes, rhoptries and dense granules [21]. Using crosses between type I, II and III it was determined that strain differences in virulence in mice can be largely explained by the exact allelic combination of the *Toxoplasma ROP18* and *ROP5* genes [19], [20], [22]–[25]. ROP18 and ROP5 are a secreted rhoptry kinase and pseudokinase, respectively, that cooperatively inhibit the murine IFNγ-induced immunity-related GTPases (IRGs) [26]–[29]. These IRGs can vesiculate the parasitophorous vacuole membrane (PVM) which ultimately leads to the destruction of the parasite inside [30]. *Toxoplasma* polymorphic secreted effectors that modulate host immune signaling pathways also play a role in the differences in virulence between type I, II and III strains. For example, the secreted rhoptry kinase ROP16 from type I and III, but not from type II, is involved in constitutive activation of the STAT transcription factors [22], [31] while the secreted dense granule protein GRA15, from type II, but not from type I and III, mediates high levels of NFκB activation [32]. Expression level differences in ROP38, another secreted rhoptry kinase, mediates strain differences in gene activation along the MAP kinase pathway [33]. Thus, the precise allelic combination of *Toxoplasma* secreted effectors likely determines how distinct *Toxoplasma* strains modulate the host immune response. GRA15 and ROP16 effects can be observed in human, mouse and chicken cells [32], [34], suggesting that *Toxoplasma* effectors that modulate host cell transcription might have evolved to target conserved host proteins.

Part of the variability in disease outcome in human infections may also be tied to strain type. The few severe congenital toxoplasmosis cases in Europe seem to be caused by atypical strains (not type I, II or III) [35]. In North America the incidence of more severe human congenital infections is higher compared to Europe and this seems to be associated with a higher prevalence of non-type II strains [9], most likely type 12 strains as these are also prevalent in North America. North American atypical strains have also been associated with severe ocular disease in non immunosuppressed patients [36]. In South America, where *Toxoplasma* strain diversity is high, congenital and ocular toxoplasmosis are more prevalent compared to Europe and more often associated with severe symptoms [4], [37]. Some strains from French Guiana can cause severe disease and even death in non immunosuppressed individuals [38]–[41].

Most of the data currently available on *Toxoplasma*-host cell interactions were obtained using the canonical types I, II or III strains, and little is known about how the atypical strains interact with and modify host cells. Because these atypical strains are correlated with more severe disease manifestations, it is important to determine how they differ from the canonical strains in modulating the host cell. To identify host cell signaling pathways uniquely modulated by atypical *Toxoplasma* strains we infected macrophages with 29 different strains, representing world-wide diversity, and obtained global transcriptional profiles of both the host and parasites. The parasite transcriptomes showed large differences in gene expression, including expression of host effectors, which correlated with differences in expression of transcriptional regulators such as the Apetala-2 (*AP2*) factors. Combining genome and transcriptome data also identified that some strains had large structural variations that had an impact on gene expression levels. Analysis of the host cell transcriptomes revealed activation of several signaling pathways in a strain-specific manner. For example, only a few atypical strains induce macrophage type I interferon production, which was associated with parasite killing, independent of interferon gamma, that leads to the release of parasite ligands that trigger intracellular Toll-like receptors and cytoplasmic nucleic acid sensors. This resource provides insight into the host response to a large variety of *Toxoplasma* strains and, together with the SNPs dataset we recently published for these strains [18], can form the basis for the discovery of novel *Toxoplasma* effectors.

## Results

### Co-regulated host and parasite gene clusters are enriched in functional annotation

**Figure 1:**
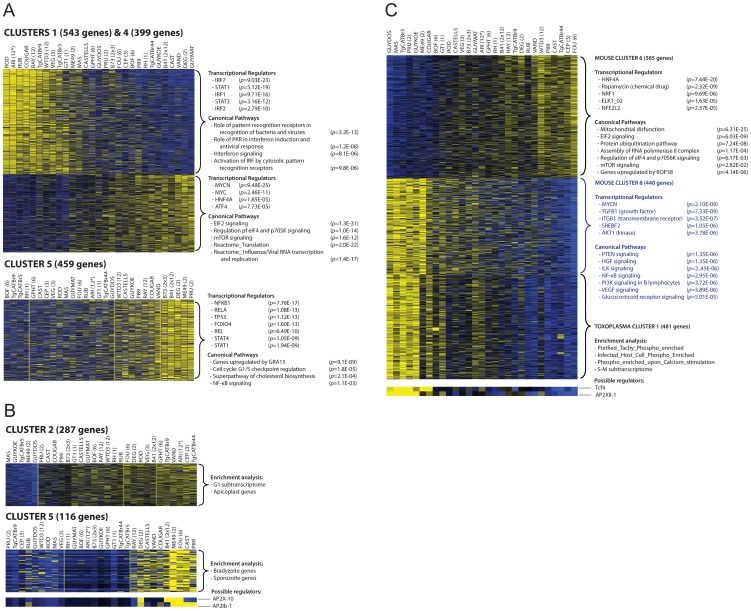
Transcriptome analysis of host cells infected with 29 *Toxoplasma* strains reveals clusters of co-regulated genes enriched in functional annotation. (A–B) Representative heat maps are shown of differentially expressed mouse (A) or *Toxoplasma* gene clusters (B). Results of the analysis of enrichment in functional annotation using DiRE, GSEA, and Ingenuity Pathway Analysis are shown. (C) Example of clusters of co-regulated mouse genes that display high correlation with a cluster of *Toxoplasma* co-regulated genes. Other clusters are in Figures S2, S3 and details of the genes present in each cluster can be found in Table S1.

We used RNA-sequencing (RNA-seq) of poly-adenylated RNA, obtained after infection of primary murine bone-marrow derived macrophages with 29 different *Toxoplasma* strains for 20 h, to capture the mouse and *Toxoplasma* transcriptomes. We detected 51% (13,179) of murine genes and 93% (8,138) of *Toxoplasma* genes (RPKM>1 in at least 1 sample) (Table S1 available online). Overall *Toxoplasma* induced macrophage gene expression correlated well with Affymetrix microarrays performed on these cells with some of the same strains (Protocols S1–2) but with significantly more genes detected by RNA-seq and a larger dynamic range (Figure S1). We focused on 2,451 differentially expressed *Toxoplasma* (RPKM>10 in at least 1 sample and at least 4-fold different between 2 samples) and 5,016 differentially regulated host genes (RPKM>5 in at least 1 sample and at least 2-fold different between any 2 samples). To identify co-regulated host and parasite genes we clustered the macrophage and parasite gene expression profiles and also clustered the experiments (strains) to identify parasite strains that modulate host gene expression similarly or that have similar parasite gene expression profiles. Clustering the macrophage and parasite expression data independently into 9–13 major clusters each adequately partitioned the data into co-regulated gene clusters (Figure 1; Figures S2, S3). Most clusters had significant functional enrichment in biological processes and the promoters of the genes in the clusters were significantly enriched for transcription factor binding sites (TFBS) indicating that these clusters represent differential modulation of host/parasite signaling pathways by the different *Toxoplasma* strains. The induction of host genes in some clusters correlated with strain type: for example, cluster 1 is induced by all type 12 strains, cluster 5 is mainly induced by type II strains or strains that have part of the type II genome and cluster 7 and 9 by type 6 strains, possibly indicating host cells responding to genetic differences between strain types. Genes in the other clusters were differently regulated by strains of the same clonal haplogroup (*e.g*, ME49 and DEG for cluster 6 and 8) possibly indicating host cells responding to epigenetic differences between strains.

### Multiple factors determine gene expression differences among *Toxoplasma* strains

Gene expression differences among the *Toxoplasma* strains could have multiple causes. For instance, the specific host cellular state can influence *Toxoplasma* gene expression [42] and *Toxoplasma* secreted effectors could modulate host gene expression resulting in an altered host cell environment to which it subsequently responds. Indeed we detected strong correlations between some host and *Toxoplasma* gene expression clusters (Table S2). For example, host gene cluster 6 and 8, which are negatively correlated (R = −0.93), have a significant negative (R = −0.86) and positive (R = 0.85) correlation with *Toxoplasma* gene cluster 1, respectively (Figure 1C). Host cluster 6 was enriched for genes and pathways that were previously shown to be modulated in human fibroblasts by the *Toxoplasma* secreted kinase ROP38 (Figure 1) [33]. Roos and colleagues have shown that ROP38 overexpression has a profound effect on host cell gene expression and modulates host genes enriched in mitochondrial function, metabolic processes and proteasome function and genes modulated by ELK1 [33], a transcriptional activator belonging to the ternary complex factor (Tcf) family. This is consistent with the functional enrichments seen for cluster 6, suggesting that this cluster might be regulated by strain differences in ROP38 expression and that *Toxoplasma* might be subsequently responding to the different environment established by ROP38. Consistent with this notion the expression level of *ROP38* is highly variable among *Toxoplasma* strains ranging from low expression in RH (RPKM = 1.1) to extremely high expression in CEP (RPKM = 505) and positively correlates with cluster 6 (R = 0.49) and negatively correlates with cluster 9 (R = −0.51).

Differences in expression levels of *Toxoplasma* transcription factors could also affect a large number of genes. To determine the putative *Toxoplasma* regulators that could modulate the gene expression clusters we used Genomica [43], which takes a set of putative regulators (Table S1) and assumes that regulators are themselves transcriptionally regulated, so that their expression profiles provide information about their activity level. Although there are many exceptions to this assumption (*e.g.* activation by phosphorylation of signaling transducers, nuclear translocation of transcription factors upon activation or activation by non-variable polymorphic transcriptional factors), the expression level of specific AP2 factors, which are known to be involved in *Toxoplasma* gene expression, correlated highly with the expression of *Toxoplasma* genes in different clusters. For example *Toxoplasma* cluster 5 and 6 are enriched in genes expressed in the bradyzoite stage and each of these clusters correlate highly with AP2 factors that are known to be upregulated during bradyzoite development [44] (AP2X-10 and AP2Ib-1 for cluster 5 and AP2IX-1 and AP2-IX-9 for cluster 6, Figures 1B; S3 and Table S1). Other non-AP2 putative *Toxoplasma* gene expression regulators were better predictors of the expression levels of *Toxoplasma* genes in different clusters (Table S1).

**Figure 2:**
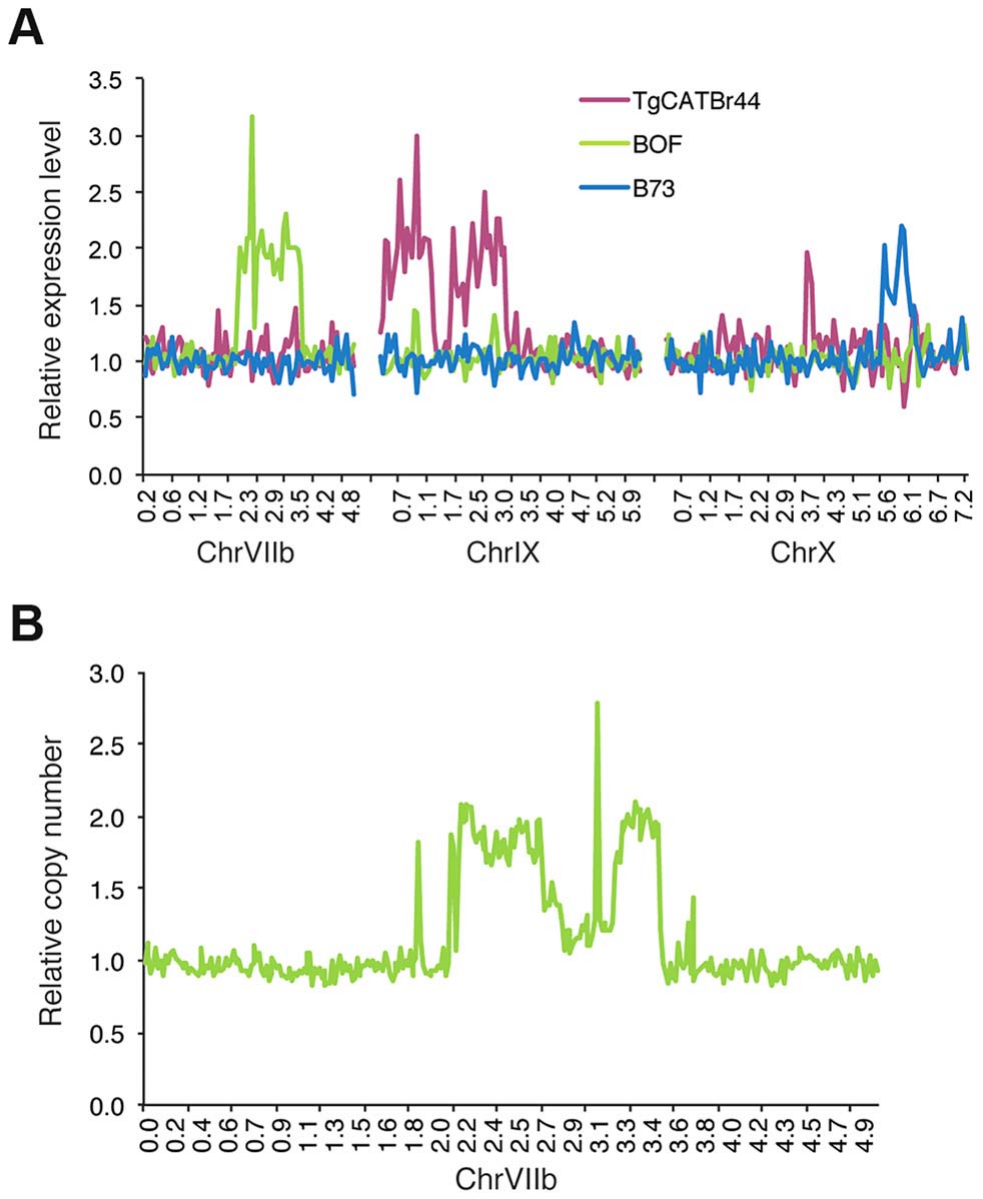
Genome-wide expression analysis uncovers strain-specific structural variations in *Toxoplasma*. (A) Average relative gene expression of strains TgCATBr44, BOF and B73, as calculated by dividing that strain's gene expression by the median gene expression of all strains, plotted against the position in chromosomes VIIb, IX and X. (B) Relative number of reads obtained from BOF sequenced genome and mapped to ChrVIIb averaged over 15 kb intervals plotted against chromosomal positions.

To identify potential large structural variations that also might influence *Toxoplasma* gene expression levels we made genome-wide graphs of the relative gene expression values for each strain against the position on the genome. This revealed that BOF, B73 and TgCatBr44 each had a large genomic region with on average twice the median expression value (Figure 2A; BOF [chrVIIb; 2.1–2.75 Mb and 3.2–3.52 Mb], B73 [chrX; 5.5–6.2] and TgCatBr44 [chrIX; 0.3–1.28 Mb and 1.7–3.2 Mb]), suggesting a large duplication. We previously sequenced the genome of BOF [18] and we used this data to confirm the putative structural variation identified using relative gene expression levels. To do this we plotted the relative number of reads mapped to chrVIIb averaged over 15 kb intervals. Indeed this identified two regions (2.1–2.75 and 3.2–3.52 Mb) on BOF chrVIIb that have on average twice as many reads compared to the rest of the chromosome and are therefore likely duplicated (Figure 2B). No such duplicated regions were detected in GPHT and FOU, even though these strains are highly related to BOF [18] and together constitute the clonal type 6 haplogroup, showing that even within a clonal lineage there can be significant variation leading to differences in gene expression levels.

### Allelic and expression differences in the *GRA15* gene determine strain differences in NFκB activation

Mouse gene expression cluster 5 contained 459 genes that were mainly upregulated in cells infected with type II (ME49, DEG and PRU), B41 and B73 strains (Figure 1A). Functional enrichment analysis showed that this cluster was enriched in genes activated by NFκB transcription factors (NFκB1, RelA) and pathway analysis also indicated enrichment in pathways consistent with NFκB activation. Type II, but not type I/III, strains strongly activate NFκB-dependent gene expression, which is due to the polymorphic secreted dense granule protein GRA15 [32]. Indeed, genes in cluster 5 were also highly enriched in genes that are upregulated by GRA15. To investigate the upregulation of NFκB regulated genes and strain type further we sequenced *GRA15* from all strains used in this study. This showed that type II, B41 and B73 strains share the same *GRA15* allele (GRA15_II_), which is shorter than the other alleles because of a 255 bp deletion (Figure S4 and S5). As expected, activation and translocation of the NFκB p65 subunit to the host cell nucleus can only be observed in cells infected with parasites strains expressing *GRA15_II_*, and is abolished in type II parasites in which *GRA15* gene was knocked out (Figure S6).

**Figure 3:**
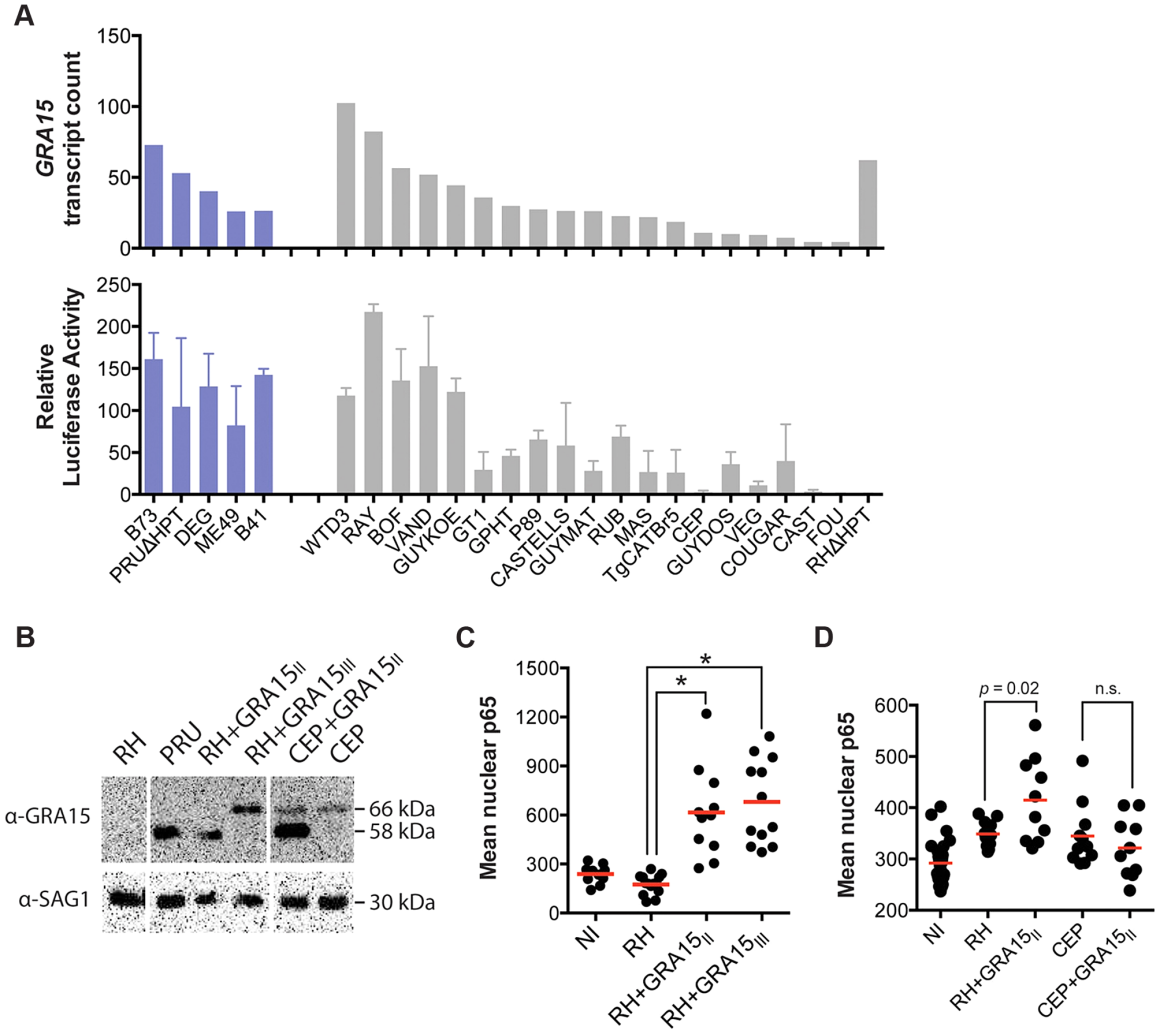
*Toxoplasma* NFκB activation depends on both the expression levels and specific allele of dense granule protein GRA15. (A) Top: *GRA15* gene expression levels as determined by high-throughput RNA sequencing. Bottom: Indicated *Toxoplasma* strains were used to infect HEK293 cells stably expressing luciferase gene under the control of an NFκB promoter. 20 h post infection luciferase activity was measured in cell lysates. The relative luciferase activity was calculated by normalizing the raw luminescence values to the background. Data is average of at least three independent experiments. (B) Western blot on lysates of the indicated strains using an antibody against GRA15 detects the type II GRA15 (58 kDa) and the type I/III GRA15 (66 kDa). SAG1 antibody was used as parasite loading control. (C–D) Human foreskin fibroblasts where infected with the indicated strains of *T. gondii* for 24 hours, fixed and stained with α-NFκB (p65). Levels of nuclear p65 were quantified using NIS-Elements software (Nikon). NI = non-infected. Data is representative of two independent experiments.

Nevertheless, we observed that several atypical strains, for example VAND, RAY, WTD3, GUY-KOE, P89, CASTELLS and COUGAR, all of which express a full length copy of *GRA15*, were also able to induce a less intense but significant NFκB response (Figure 1A), suggesting that other parasite factor(s) may play a role in controlling NFκB activation. To further investigate this, we infected an reporter cell line expressing a luciferase gene under the control of NFκB transcription factor response elements with the different *T. gondii* strains and observed that strains with higher expression of *GRA15*, including VAND, WTD3, RAY and GUY-KOE, induced higher activation of the reporter regardless of the *GRA15* allele they express (Pearson correlation between GRA15 expression level and luciferase activity = 0.7, Figure 3A). The exception was RH, which expresses a truncated non-functional form of GRA15 due to a premature stop codon [32]. This suggests that expression levels of GRA15 may also be significant in controlling NFκB activation. To test this hypothesis we designed transgenic RH parasites overexpressing either the type II (58 kDa) or type III (66 kDa) GRA15. Levels of transgenic expression of GRA15 were similar in both parasites (Figure 3B). Using immunofluorescence analysis of infected human foreskin fibroblasts we observed that transgenic overexpression of the type III copy of *GRA15* in RH parasites also leads to efficient NFκB activation (Figure 3C). Still, not all strains with high expression levels of *GRA15* (*e.g.* BOF) induced a strong pro-inflammatory response (Figure 1A) or are able to induce nuclear translocation of NFκB subunit p65 (Figure S6). Taken together, our data suggest that although GRA15_II_ is the main activator of NFκB, high expression levels of different *GRA15* alleles can also induce pro-inflammatory responses in infected host cells, possibly through activation of different NFκB subunits.

### The host cell pro-inflammatory response is modulated by multiple *Toxoplasma* secreted factors

**Figure 4:**
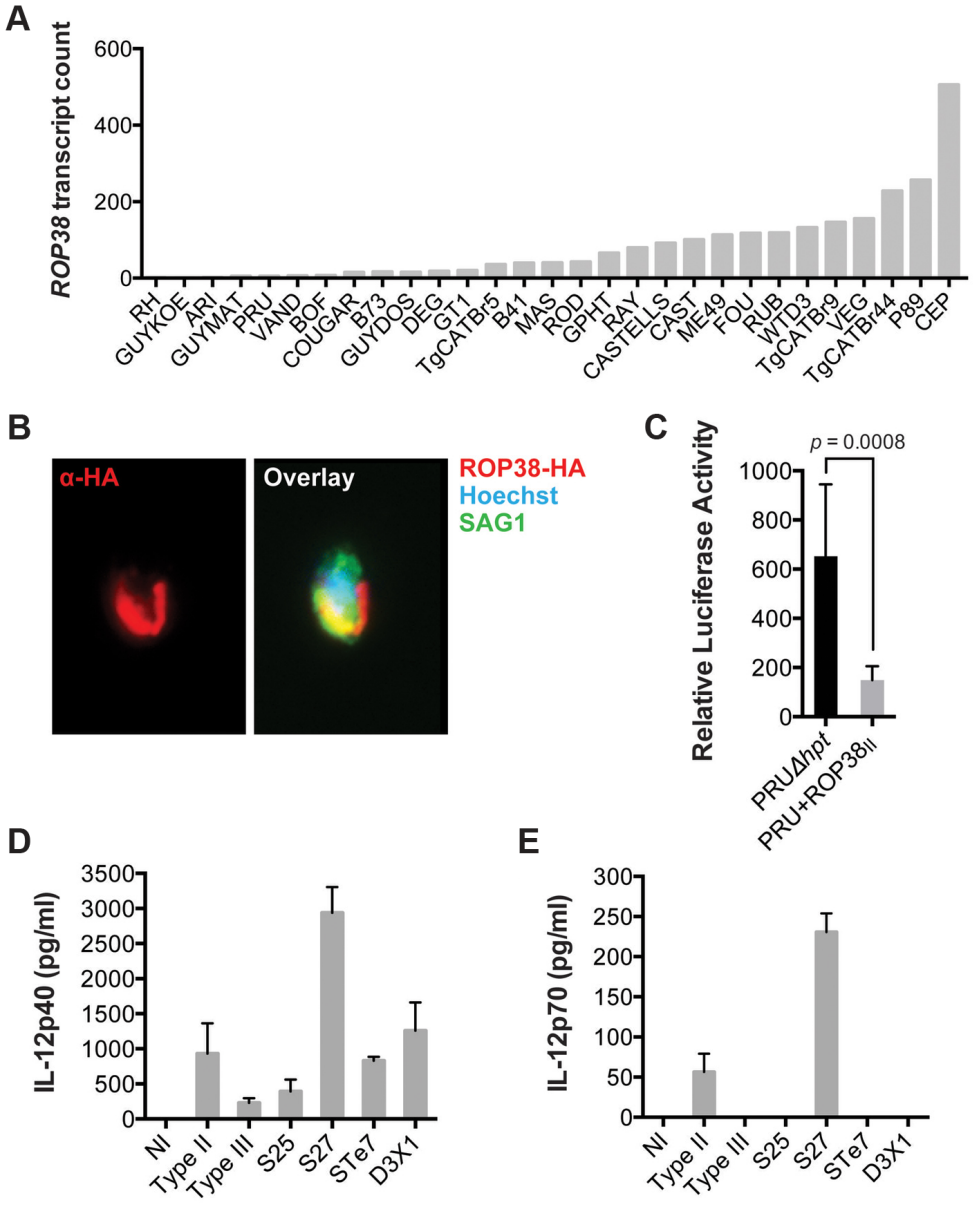
ROP38 inhibits NFκB activation. (A) Expression level of ROP38 in both control parasites (PRU*Δhpt*) and PRU transgenically expressing ROP38_II_, as detected by RNA sequencing. (B) Type II (PRU) parasites overexpressing HA-tagged ROP38 were used to infect monolayers of HFFs. After 3 h cells were fixed and stained with anti-HA to detect ROP38 (red), anti-SAG1 to detect parasites and Hoechst to detect parasite nucleus (blue). (C) HEK293 cells stably expressing luciferase under control of NFκB canonical promoter elements were infected with the indicated strains of *Toxoplasma*. 20 h post infection luciferase activity was measured in cell lysates. Data depicted is the combination of three independent experiments. (D–E) BMDM were infected for 24 h with a type II, III and F1 progeny from IIxIII crosses that contained type II *GRA15* and *ROP16* alleles (S25, S27, STE7, D3X1) and IL-12p40 (D) and IL12p35 (E) levels were detected in the supernatant using ELISA. Bars = standard error. Similar results were obtained in at least two independent experiments.

We have previously shown that GRA15 induction of an NFκB-dependent pro-inflammatory response can be antagonized by the activity of *Toxoplasma* polymorphic rhoptry kinase ROP16 [45]. Recently, ROP38, a rhoptry protein that is highly expressed in type III parasites relative to types I/II, was shown to downregulate several host cell responses, including cytokine production, induced during infection [33]. Indeed, we observed that *ROP38* expression was highly variable in the different strains, with the highest expression observed in the type III strain CEP (Figure 4A). We previously observed by microarray analysis that CEP expressing GRA15_II_ induced significantly lower levels of NFκB-regulated genes compared to type I+GRA15_II_ [32]. We therefore wanted to test whether ROP38 can dampen pro-inflammatory responses activated by GRA15_II_. Indeed, only RH-GRA15_II_, but not CEP-GRA15_II_, induced nuclear translocation of NFκB subunit p65 (Figure 3D) even though both transgenic strains expressed similar levels of GRA15 (Figure 3B). To test if the higher expression of ROP38 observed in CEP could be responsible for this phenotype, we engineered type II (PRU) parasites to overexpress a transgenic copy of HA-tagged ROP38 (Figure 4B). Compared to the control type II parasites, activation of the NFκB reporter cell line is markedly reduced in cells infected with parasites overexpressing ROP38 (Figure 4C).

We have also previously shown that GRA15_II_ induces and ROP16_I/III_ represses IL12p40/p70 production by infected macrophages [32], [45]. Because NFkB activation is required for IL12 production we wanted to confirm that other genes, besides GRA15 and ROP16, could play a role in strain differences in IL12 induction. We therefore infected BMDM with F1 progeny derived from IIxIII crosses that have both type II *GRA15* and *ROP16* alleles (S25, S27, STE7, D3X1) and assessed production of IL12p40 and IL12p70. Even though both the S25 and S27 strains have *ROP38* alleles derived from the parental type II strain (data not shown), only the type II and S27 infection led to the production of high levels of IL12p70 (Figures 4D–E), indicating that besides *GRA15* and *ROP16* other genes play a role in the strain-specific induction of IL12 secretion. Thus, although GRA15_II_ is the main parasite factor controlling NFkB-dependent host cell responses, the expression level of other *GRA15* alleles and other *Toxoplasma* secreted proteins, including ROP16 and ROP38, also affect host cell inflammatory responses. This is reflected in mouse expression clusters 7 and 9, which contain genes that are mainly induced by strains that do not activate a strong inflammatory response (type 6, RH, CAST and CEP).

### Effector sequence versus activity correlations can pinpoint critical residues that determine strain differences in activity

**Figure 5:**
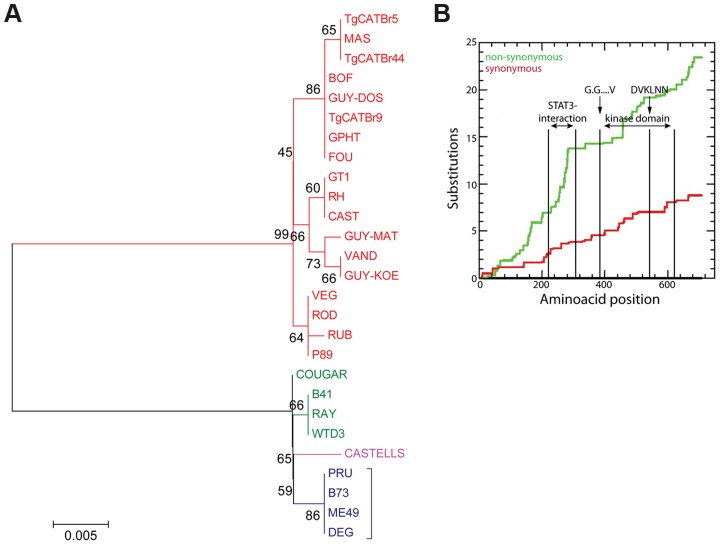
Evolutionary analysis of ROP16. (A) Evolutionary bootstrap consensus tree of ROP16 was inferred from 500 replicates using the Neighbor-Joining method. The percentages of replicate trees in which the associated taxa clustered together in the bootstrap test (500 replicates) are shown next to the branches. The tree is drawn to scale, with branch lengths in the same units as those of the evolutionary distances used to infer the phylogenetic tree. The evolutionary distances were computed using the Poisson correction method and are in the units of the number of amino acid substitutions per site. Analyses were performed in MEGA5 software. (B) Cumulative behavior, codon by codon, of the average synonymous and non-synonymous substitutions rates of GRA15 alleles performed using the Synonymous Non-synonymous Analysis Program (SNAP, [83]). STAT3 interacting domain and kinase domain are indicated.

Once a *Toxoplasma* effector that modulates strain-specific regulation of host signaling pathways is identified it is often unclear what exactly determines the strain specificity of that activity. For example type I, II and III all have similar expression levels of the polymorphic rhoptry kinase ROP16 but only ROP16 from types I and III constitutively activate STAT3 and STAT6 [22]. By making chimeric ROP16 from type I and II and subsequent individual substitutions it was determined that a single leucine to serine substitution at position 503 (L→S503) in the kinase domain of type II ROP16 (ROP16_II_) is responsible for its significantly reduced kinase activity for its STAT3 substrate [22], [46], [47]. To determine whether data from multiple strains could have predicted that the L→S503 is the critical difference that determines strain differences in ROP16 activity we used our RNA-seq data to assemble the *ROP16* allele for each strain, perform evolutionary analysis (Figures 5A and S7) and determine for each strain if it could constitutively activate STAT6, a reliable read out for ROP16 activity. All strains with a non-type II *ROP16* allele constitutively activated STAT5 and STAT6 (Figures S8, S9). COUGAR, which constitutively activates STAT6, has a ROP16 that only differs at two positions from type II ROP16, it has an alanine at position 502 and a leucine at 503. Thus, sequence versus activity correlations of a polymorphic effector using data from a large collection of strains can directly pinpoint the crucial residue(s) that mediate the strain differences in the activity of the effector. We also observed higher rates of non-synonymous/synonymous (NS/S) polymorphisms at the STAT3-interaction site [22], [46], [47] and part of the kinase domains of ROP16 (Figure 5B). The fact that ROP16 from all non-type II strains is active, with the exception of B73 (Figures S8, S9, data not shown), which is an F1 from a cross between type II and type III [18], suggests that the ROP16_II_ L→S mutation is a recent event that occurred after divergence of type II from the closely related WTD3, B41 and RAY type 12-like strains.

### *Toxoplasma* atypical strains activate type I interferons

**Figure 6:**
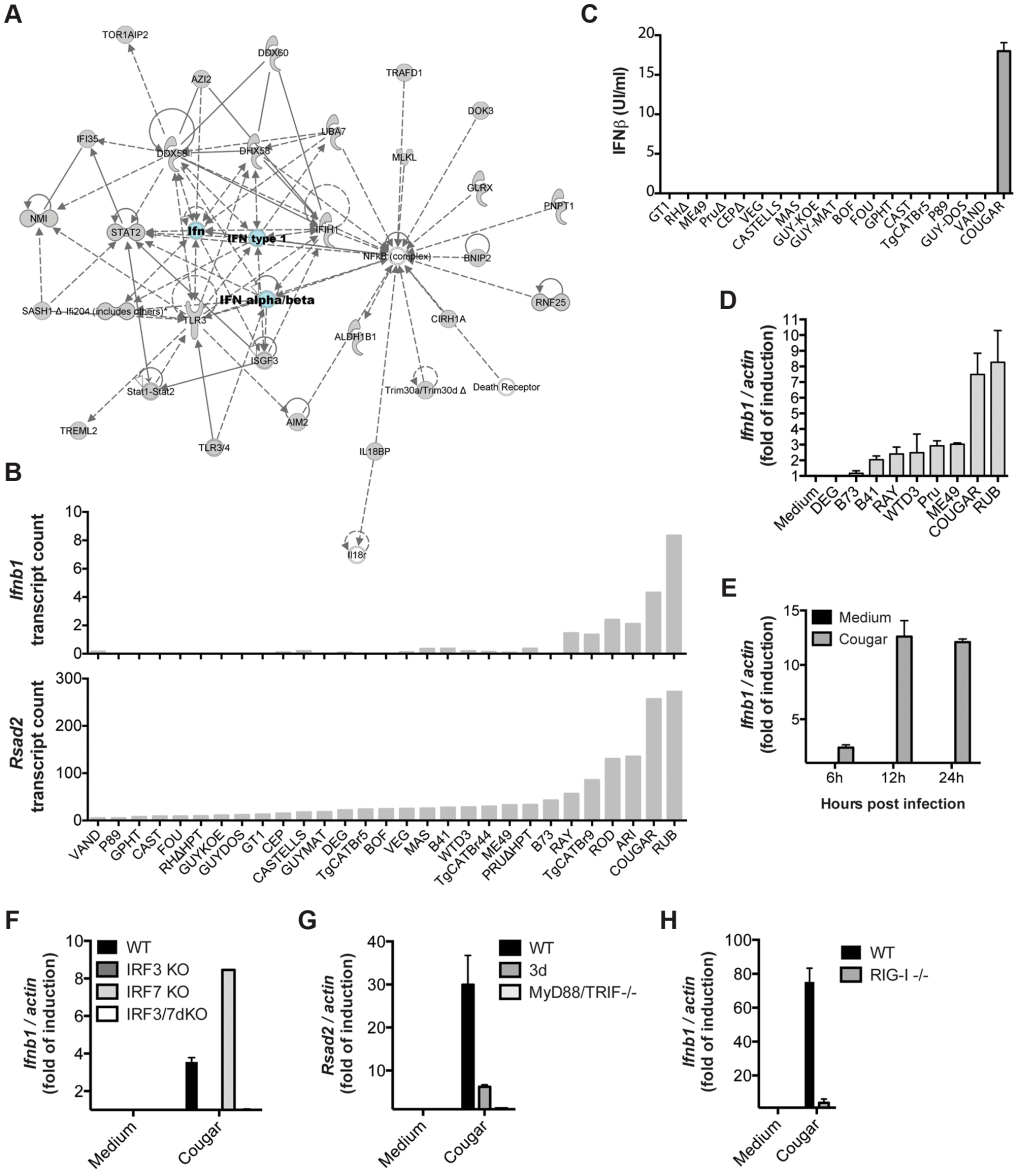
Strain-specific activation of the type I interferon signaling pathway. (A) Ingenuity network based on the clustered genes is centered on IFNβ. (B) Expression levels of *Ifnb* (top) and *Rsad2* (bottom) in BMDMs infected with the indicated strains of *T. gondii* for 20 h as determined by RNA sequencing. (C) Secretion of IFNβ protein by BMDMs into culture supernatants after infection with the indicated strains was detected 24 h post-infection by ELISA. (D) BMDMs were infected with indicated *Toxoplasma* strains for 24 h, and subsequently qPCR was used to detect *Ifnb1*. Relative expression levels were calculated by normalizing against actin expression. (E) BMDMs were infected with COUGAR and RNA isolated at the indicated time points. qPCR was used to detect *Ifnb1*. (F–H) Wild-type, IRF3, IRF7 and IRF3/IRF7 knockout immortalized macrophages (F), Unc93b1 triple-deficient mutant (3 d) and MyD88/TRIF double knockout immortalized macrophages (G), or wild-type and RIG-I knockout MEFs (H) were infected with COUGAR and qPCR was used to detect *Ifnb1*, *Rsad2* and *actin* expression levels. Data is representative of three (C–D) or two (E–H) experiments yielding similar results.

Among the novel pathways we identified, we observed that most type 12 *Toxoplasma* strains, and some other atypical strains, induced a cluster of 543 genes (Cluster 1 in Figure 1) that are enriched in components of the type I interferon signaling pathway, including *Ifnb1*, the family of RNA helicases *Ddx58* (RIG-I), *Dhx58* (LGP2) and *Ifih1* (MDA5), several interferon inducible proteins, including *Ifitm3* and *Ifi35*, and immunity-related GTPases (IRGs). Upstream regulator analysis indicated that the Interferon Regulatory Factors (IRF1, IRF2, IRF7) were the most likely regulators of this cluster (Figure 1A). Pathway and network analyses of these genes also revealed enrichment for genes involved in IFNβ signaling (Figures 1A and 6A). Another cluster (Cluster 4) containing 399 genes had a high negative correlation with the IFN cluster (R = −0.93). This cluster was highly enriched for genes involved in EIF2 signaling and translation (Figure 1A). It is likely that type I IFN production led to activation of protein kinase R (PKR), which can phosphorylate EIF2A and thereby block translation.

Only a few atypical *Toxoplasma* strains were capable of inducing high levels of *Ifnb* and *Rsad2* (an IFNβ induced gene also called viperin) expression, the most potent inducers being RUB and COUGAR (Figure 6B). To validate our RNA-seq data, we infected BMDMs with several *Toxoplasma* strains and measured interferon beta in the supernatants 24 hours post infection. Both IFNβ protein and IFNβ transcripts were produced by cells infected with COUGAR and RUB, but not other canonical or atypical strains tested (Figures 6C–D). Time course experiments showed that IFNβ transcripts can be detected as early as 8 h post infection, peaking at 12 h (Figure 6E and data not shown).

### *Toxoplasma* induction of type I interferon is dependent on IRF3 and endosomal TLRs or RIG-I

Transcriptional regulation of type I interferons by viral pathogens depends on the activity of either IRF3 and/or IRF7 [48]. To investigate whether *Toxoplasma* induction of IFNβ was also dependent on these host cell transcription factors, we infected immortalized macrophages, derived from wild type or mice deficient for different IRFs, with the ‘activating’ strain COUGAR. *Ifnb* transcription was completely abolished in infected cells lacking IRF3, but not in cells lacking IRF7 (Figure 6F), showing that the IRF3 transcription factor is essential for parasite-induced IFNβ production. As expected, single IRF knockout cells were still responsive to synthetic double stranded DNA poly d(A)∶d(T), but cells missing both IRF3 and IRF7 were unresponsive (Figure S10A). To test which host cell signaling pathway was being used by atypical *Toxoplasma* strains to activate IRF3, we infected wild type, Unc93B1 3 d mutant (which are defective in intracellular TLR trafficking and activation) or MyD88 and TRIF double knockout immortalized macrophages with COUGAR and performed qPCR analysis for *Rsad2* 20 h post infection. *Rsad2* transcription was completely abolished in infected MyD88/TRIF dKO macrophages (Figure 6G) and strongly reduced in 3 d cells, suggesting that endosomal Toll-like receptors are essential for parasite-induced IFNβ production in macrophages. Importantly, the absence of TLR signaling did not affect the ability of macrophages to produce IFNβ in response to transfected double stranded DNA (Figure S10B). Nevertheless, we observed that in murine fibroblasts, which express much lower levels of TLRs than macrophages [49], *Ifnb* transcript levels after both COUGAR infection or poly d(A)∶d(T) treatment are dependent on the cytoplasmic nucleic acid receptor RIG-I (Figure 6H; Figure S10C). Taken together our data show that IFNβ induction by atypical *Toxoplasma* strains is dependent on activation of different nucleic acid sensing pathways depending on host cell type.

### *Toxoplasma* also induces type I interferon production in human cells

**Figure 7:**
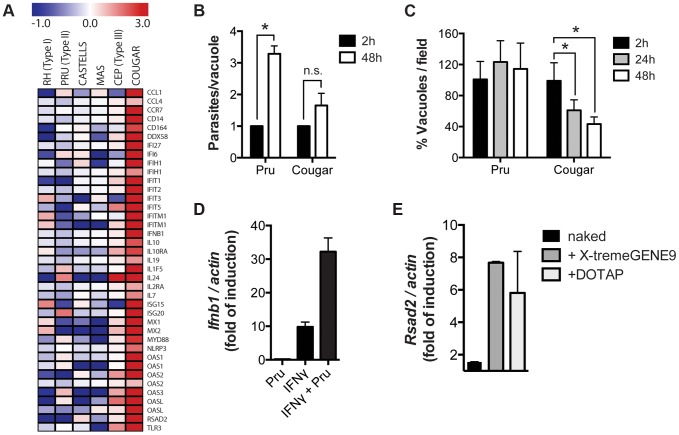
Killing of intracellular parasites is associated with IFNβ production. (A) Microarray analysis of HFFs shows differentially expressed host genes belonging to the type I interferon pathway (rows) induced during infection with different *T. gondii* strains (columns). (B–C) HFFs monolayers were infected with either PRU or COUGAR parasites for two hours, and subsequently washed to remove extracellular parasites. At the indicated time points cells were fixed, permeabilized, and stained with anti-SAG1 and anti-GRA7 to visualize parasites and vacuoles respectively. Number of parasites per cell (B) and vacuoles per field (C) was determined by counting at least 60 infected cells and 5 microscope fields per condition. Error bars represent standard error. Data is representative of three independent experiments. * = *p*<0.05. (D) HFFs monolayers where either pre-treated with IFNγ 100 u/ml for 24 hours or left untreated, and subsequently infected with *T. gondii* (PRU strain). After 20 hours of infection total RNA was isolated and expresion levels of *Ifnb1* was determined by qPCR. Data is representative of experiments done two times. (E) Immortalized murine macrophages were stimulated overnight with 2.5 µg/ml of *T. gondii* genomic DNA or Poly(dA∶dT) either naked or conjugated with transfection reagents that delivers nucleic acids to the cytoplasm (X-tremeGENE9) or endosomal compartments (DOTAP). Subsequently total RNA was isolated and expression levels of *Ifnb1* was determined by qPCR. Data is representative of two independent experiments.

To determine if COUGAR also induced type I IFN production in human cells we performed transcriptional analysis of HFFs infected with either canonical or atypical *Toxoplasma* strains. We observed that COUGAR could also induce a strong IFNβ signature in human cells, whereas neither the canonical strains nor atypical strains CASTELLS and MAS did (Figure 7A). Since we observed that IFNβ induction is dependent on activation of nucleic acid sensors, we hypothesized that strains capable of inducing IFNβ are somehow being destroyed by the host cells, releasing both parasite DNA and RNA that are then detected by either TLRs or RLRs. To test this, we infected HFFs monolayers with a non-activating type II (PRU strain) or the activating COUGAR strain. As the typical lytic cycle of PRU and COUGAR takes about 72 hours, we chose 24 h and 48 h time points to evaluate parasite growth prior to lysis of host cells. Cells were fixed, stained with antibodies against GRA7 and SAG1 to visualize both parasite vacuoles and parasites respectively. As expected, in monolayers infected with PRU, the number of vacuoles per field remained constant throughout the experiment, while the number of parasites per vacuole increased due to parasite division (Figures 7B–C). In cells infected with COUGAR, we observed a marked reduction in the number of vacuoles per field, suggesting that the host cells were destroying the vacuoles, and the increase in number of parasites per vacuole was negligible (Figures 7B–C).

Previously, we showed that in IFNγ-stimulated MEFs, type II strain vacuoles are rapidly coated with IRGs after which they are destroyed [28], potentially releasing parasite DNA and RNA into the host cell cytoplasm. To test if parasite killing can induce the production of type I IFN, we infected MEFs that were pre-stimulated with IFNγ with a type II strain. Indeed IFNγ-stimulated type II infected cells produced much higher *Ifnb* levels compared to IFNγ-stimulated or control cells (Figure 7D). Moreover, transfection of *Toxoplasma* DNA into host cells was sufficient to induce the upregulation of the type I IFN induced gene *Rsad2* (Figure 7E).

**Figure 8:**
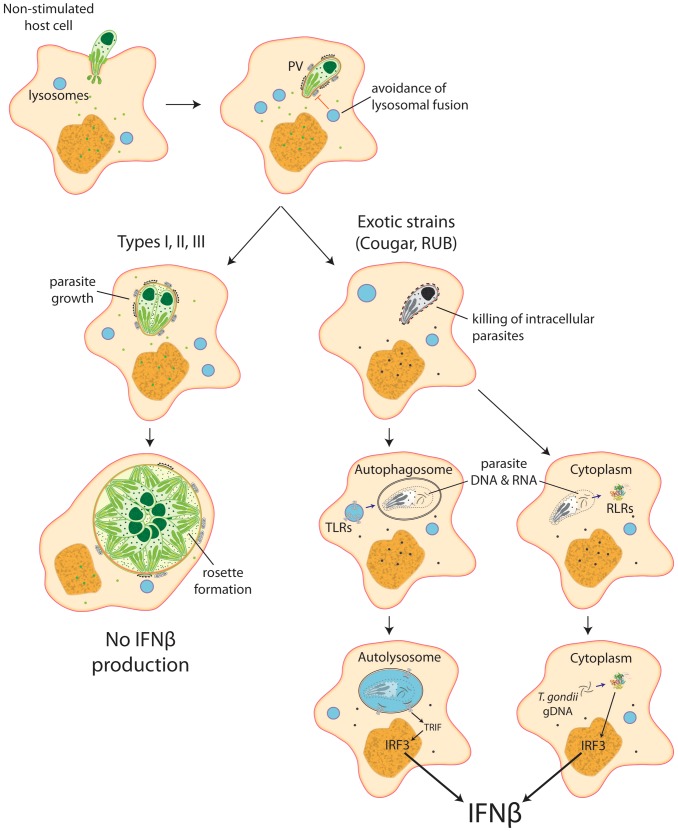
Schematic model depicting induction of type I interferons by *Toxoplasma*. Most parasite strains, when infecting resting, non-stimulated host cells, establish themselves in a parasitophorous vacuole that is permissive to parasite growth and do not induce IFNβ. Nevertheless, some atypical strains, like COUGAR and RUB, are killed right after formation of the parasitophorous vacuole, releasing parasite nucleic acid that can be captured by autophagossomes or released into the host cell cytoplasm, where it can interact with either Toll-like receptors or RIG-I-like receptors resulting in production of IFNβ.

Our data show that intracellular killing of *T. gondii* results in the release of parasite nucleic acids that lead to activation of either TLR or RLR receptors, triggering a signaling cascade that activates IRF3 and culminates in the production of IFNβ (Figure 8). This suggests that ‘resting’ cells can eliminate intracellular parasites through a novel, IFNγ-independent killing mechanism, and some atypical strains of *Toxoplasma* are more susceptible to this killing mechanism, resulting in increased induction of the type I IFN response.

## Discussion

In South America both congenital and ocular toxoplasmosis are more prevalent compared to Europe and more often associated with severe symptoms [4], [37]. There is some evidence that this is associated with atypical strains (not type I, II or III), mainly present in South America. Some strains from French Guiana can cause severe disease and even death in non immunosuppressed individuals [38]–[41]. Also in North America atypical (non type I/II/III) strains have been associated with more severe disease symptoms [36]. However, it is currently unknown if these atypical strains differ in their interaction with host cells. Here we provide the complete murine macrophage and *Toxoplasma* transcriptomes after infection with 29 different *Toxoplasma* strains. We have identified clusters of co-regulated macrophage and parasite genes that are enriched for functional annotation. We expect that this data will form the basis for exploring the molecular basis for the differential regulation of these gene expression clusters.

As an example we explored a cluster of co-regulated host genes that are enriched in genes regulated by type I IFN and show that only a small number of atypical strains activate the type I IFN response. We show that the type I IFN induction by these strains is associated with the killing of parasites in non-stimulated cells and the recognition of DNA/RNA by endosomal and/or cytoplasmic pattern recognition receptors.

The combination of *Toxoplasma* secreted effectors that leads to optimal fitness in one host is unlikely to be optimal in all of *Toxoplasma*'s hosts and if strains end up in the wrong host they might cause disease by either causing pathological inflammation or by overwhelming the host. Indeed, different host species display large differences in susceptibility to *Toxoplasma* and different *Toxoplasma* strains often differ in virulence in the same host species suggesting that specific *Toxoplasma* strains might have adapted to certain hosts [50]–[52]. Our data indeed show large differences between strains in the modulation of host genes. One of the most consistent observations is the large difference between *Toxoplasma* strains in the activation of an inflammatory response. Here we show that this is mediated by differences in the sequence and expression level of GRA15, which can activate the pro-inflammatory transcription factor NFκB, and by differences in the activation of the type I IFN response. Other *Toxoplasma* effectors, such as the ROP16 and ROP38 protein kinases, can inhibit this inflammatory response. Therefore, the exact sequence and expression levels of GRA15, ROP16 and ROP38, determine the potential of different *Toxoplasma* strains to induce inflammation. In general the type II, type 12 and COUGAR strains are more pro-inflammatory while type I, type III and type 6 are the least inflammatory. However, even within clonal haplogroups, large differences in the induction of host gene expression exist. In North America, non-type II strains have been associated with more severe toxoplasmosis [9]. Because type 12 strains were recently shown to be prevalent in North America [11], [15]–[17], it is possible that they are the cause of severe disease.

It is interesting that our results indicate that type 12, and the closely related COUGAR strain [18], induce a gene expression profile enriched for type I IFN signaling. For the COUGAR strain, that was also confirmed in human cells. Type I interferons (IFN-α/β) are potent coordinators of antimicrobial responses, controlling both cell autonomous innate responses and T cells memory and effector functions [53], [54]. Besides their role in viral infections, they can also play an important role during parasitic infections by either controlling parasite growth through activation of intracellular killing mechanisms [55]–[60] or enhancing severity of disease caused by *Trypanosoma cruzi*, *Leishmania*, and *Plasmodium* [61]–[64]. Importantly, type I interferons can affect infection persistence [65], [66], which depends on both timing and magnitude of the interferon response [67]. We observed that the exotic strain COUGAR, is more susceptible than type II strains to IFNγ-independent killing, which leads to an early IFNβ production. This strain specific difference in timing and magnitude of type I interferon response may result in different disease outcomes. We have observed that expression of type I interferons by infected host cells is followed by induction of IRGs, which have a major role in controlling parasite growth [30]. Indeed, it has been previously shown that IFNβ can control *Toxoplasma* growth both *in vitro* and *in vivo* [68], [69]. Nevertheless, these studies were performed by pre-treating cells or animals with high doses of recombinant IFNβ, and lack an assessment of the role of type I interferons in physiological conditions. In this regard, type II strains of *Toxoplasma* were shown to stimulate type I interferon production *in vivo* by intestinal cells following oral infection with *T. gondii* cysts [70], [71], however no detailed analysis of the role of this cytokine in disease progression has been performed. Thus, even though induction of IFNβ could potentially be beneficial for the host by controlling intracellular parasite growth, it is striking that in the mouse model COUGAR parasites are significantly more virulent than type II strains [19]. It is currently unclear if this enhanced virulence was due to increased parasite burden or enhanced pathogenesis possibly due to a strong pro-inflammatory response. Because COUGAR is genetically quite different from the type II strain [18] it is possible that its enhanced virulence is unrelated to its induction of IFNβ and due to differences in other polymorphic effectors. However, in the context of recent observations that type I IFN suppresses type II IFN-triggered human anti-mycobacterial responses [72] it is plausible that the type I IFN response induced by some of the *Toxoplasma* strains could be associated with increased pathogenesis in human patients.

The simultaneous capture of both the parasite and host transcriptomes has also provided us with important insights into the crosstalk between the host and the parasite. We observed significant correlations between the expression of *Toxoplasma* and host gene clusters. It has been previously observed that the state of the host cell can modulate *Toxoplasma* gene expression. For example addition of compound 1 to host cells induces differentiation of *Toxoplasma* into bradyzoites and the knockdown of CDA1, a nucleosome assembly-related protein, demonstrated its involvement in cell cycle arrest and negative control of cell proliferation, mimicking the effects of compound 1 [42]. A potential role for the host cell cycle state in modulating *Toxoplasma* growth and differentiation has also been observed by others, for example, *Toxoplasma* spontaneously differentiates into bradyzoites upon invasion of terminally differentiated skeletal muscle and neuronal cells [73], [74]. Recently it was shown that the dense granule protein GRA16 is secreted beyond the PVM and traffics to the host cell nucleus [75], where it could potentially regulate host cell cycle. It was suggested that GRA16 downregulation of cyclin-B1 could be the main cause of the GRA16-induced G2 arrest, which has also been observed by others to be associated with *Toxoplasma* infection [76], [77]. Many of our strain-specifically regulated host gene clusters are also enriched for processes involved in cell cycle regulation suggesting that this is an important target for *Toxoplasma* effectors. For example host expression cluster 9 is enriched for host genes modulated by P53 and the CDKN1A kinase. It is also enriched in genes involved in regulation of the host cell cycle, especially G2/M checkpoint regulation. Because these are highly similar to the processes regulated by GRA16 it is tempting to speculate that strain differences in expression of GRA16 and/or sequence of GRA16 might be involved. All together, our study provides global insight of host cells and parasite transcriptional responses upon infection with both canonical and exotic *Toxoplasma* strains, and allows identification of novel strain specific host cell responses and parasite effectors.

## Materials and Methods

### Ethics statement

All experiments involving animals were in accordance with guidelines set forth by the American Association for Laboratory Animal Science (AALAS). All protocols used in this work were approved by the Committee on Animal Care (CAC #0611-063-14) at the Massachusetts Institute of Technology.

### Reagents

All tissue culture reagents were obtained from Gibco, unless otherwise indicated. Poly(dA∶dT) was obtained from Invivogen. All oligonucleotide primers were obtained from Integrated DNA Technologies.

### Mice

C57BL/6J mice were obtained from The Jackson Laboratory. Female, 6–12 weeks old mice were used in all experiments. Mice were housed under specific pathogen free conditions at Massachusetts Institute of Technology animal facility.

### Parasites

*Toxoplasma* was cultured on human foreskin fibroblasts as described previously [32]. The following *Toxoplasma* strains, with the animal and country it was originally isolated from in brackets, were used in this study: ARI (human, USA), B41 (bear, USA), B73 (bear, USA), BOF (human, Belgium), CAST (human, USA), CASTELLS (sheep, Uruguay), CEP (cat, USA), COUGAR (Cougar, Canada), DEG (human, France), FOU (human, France), GPHT (human, France), GT1 (goat, USA), GUY-DOS (human, French Guiana), GUY-KOE (human, French Guiana), GUY-MAT (human, French Guiana), MAS (human, France), ME49 (sheep, USA), P89 (pig, USA), PRU (human, France), RAY (human, USA), RH (human, USA), ROD (human, USA), RUB (human, French Guiana), TgCatBr5 (cat, Brazil), TgCatBr9 (cat, Brazil), TgCatBr44 (cat, Brazil), VAND (human, French Guiana), VEG (human, USA), WTD3 (white-tailed deer, USA) [16]. The genetic relationship among these strains was previously investigated using genome-wide SNP data [18]. Strains originating from France and French Guiana were provided by the BCR Toxoplasma (http://www.toxocrb.com). To generate PRU (ΔHXGPRT) parasites expressing the type II allele of ROP38, the ROP38 coding region and putative promoter region (1,437 bp upstream of the start codon) were amplified from PRU genomic DNA by PCR (forward, 5′-CGAGAGGGAAGCAACGTTTA-3′, reverse, 5′-TTA*CGCGTAGTCCGGGACGTCGTACGGGTA*AAATTGATGCGTTCTTATCCGACG-3′). Sequence coding for a C terminal HA tag was included in the reverse primer (denoted with italics). ROP38_II_HA was then cloned into pTKO-att [32] through LR recombination. The pTKO-att-ROP38_II_HA vector was then linearized with *Not*I (New England Biolabs, Inc.) and transfected into PRUΔHXGPRT by electroporation. Stable integrants were selected in media with 50 µg/ml mycophenolic acid (Axxora) and 50 µg/ml xanthine (Alfa Aesar) and cloned by limiting dilution. Immunofluorescence was used to confirm expression of ROP38_II_ via anti-HA staining. All parasite strains and cell lines were routinely checked for *Mycoplasma* contamination and it was never detected.

### Cells

RIG-I knockout mouse embryonic fibroblasts (MEFs), IRF3, IRF7 and IRF3/7 knockout immortalized mouse macrophages were provided by Katherine Fitzgerald (UMass Medical School). Immortalized macrophages expressing a mutant, non-functional form of Unc93B1, and MyD88/TRIF double-knockout macrophages were provided by Douglas T. Golenbock (UMass Medical School). Human foreskin fibroblasts (HFFs) and MEFs were maintained in DMEM medium supplemented with 10 mM Hepes, 100 U/ml Penicillin-Streptomycin and 10% FCS (PAA Laboratories). Immortalized macrophages were kept in DMEM supplemented with 10 mM Hepes, 100 U/ml Penicillin-Streptomycin, 1 mM Pyruvate, 10% L929 supernatant and 10% FCS.

### Bone marrow-derived macrophages infection and RNA isolation

Bone marrow-derived macrophages (BMDMs) were isolated as described [78], and were cultured in RPMI medium supplemented with 25 mM Hepes, 10 mM L-glutamine, 100 U/ml Penicillin-Streptomycin, 50 µM 2-mercaptoethanol and 10% FCS. BMDMs were seeded in 6 well plates at 70% confluency and infected with different strains of *T. gondii* at three multiplicity of infections (MOIs): 15, 10 and 7.5. At the same time a plaque assay was setup to determine the viability and real MOI of each strain. After 20 h, total RNA was isolated using the RNeasy Plus kit from Qiagen, according to the manufacturer instructions. RNA integrity, size and concentration were verified using the Agilent 2100 Bioanalyzer and samples with similar *Toxoplasma* infections rates as judged by the *Toxoplasma* ribosomal band (Figure S11) were selected for RNA-seq.

### High-throughput RNA sequencing

The RNA was processed for high-throughput sequencing according to standard Illumina protocols. Briefly, after mRNA pull down from total RNA using Dynabeads mRNA Purification Kit (Invitrogen), mRNA was fragmented into 200–400 base pair-long fragments and reverse transcribed to cDNA. Illumina sequencing adapters were added to each end, and samples were barcoded and multiplexed (four samples per lane) for sequencing on the Illumina HiSeq 2000 machine. On average 68 million 40 bp reads were obtained from each library. Reads in fastq format were mapped to either mouse (build 37.2) or *Toxoplasma gondii* (strain ME49, version 8.0) genomes using Bowtie 2.0.0 and Tophat 2.0.4. [79]. Transcripts from each sample were assembled, merged, and their expression values in FPKM were calculated using the Cufflinks package, version 2.0.2. [79]. Detailed methodology can be found in the supplemental material, and settings used are included in Table S3A–B.

### Clustering and functional annotation of *Toxoplasma gondii* strains and infected host cells transcriptome

To identify host signaling pathways modulated by *Toxoplasma* in a strain-specific manner we specifically focused on macrophage genes that had at least a 2-fold difference in expression between macrophages infected with different *Toxoplasma* strains and had FPKM≥5 in at least 1 strain. To identify differentially regulated *Toxoplasma* gene clusters we specifically focused on *Toxoplasma* genes that had at least a 4-fold difference in expression between any two different *Toxoplasma* strains and had at least a FPKM value of 10 in one strain. We used Genomica [43] to cluster the genes into 10–15 clusters of co-regulated genes. These clusters were then investigated for enrichment for functional annotation, such as enrichment for transcription factor binding sites (TFBS) in their promoters or belonging to a particular pathway using DiRE [80], GSEA [81], and Ingenuity Pathway Analysis (www.ingenuity.com). A more detailed explanation of data analysis can be found in the supplemental methods online (Protocols S1–2).

### Identification of structural variation using gene expression levels

We first identified all *Toxoplasma* protein coding genes with a median expression of >7.5 and subsequently determined the relative expression for each gene for each *Toxoplasma* strain by dividing the gene expression values, for each strain, by the median gene expression of all strains. We then made genome-wide graphs of these relative expression values for each strain by plotting the average relative expression in steps of 5 genes against the position on the genome (average position for the 5 genes). We then manually inspected the graphs for all 14 *Toxoplasma* chromosomes for each of the 29 strains for large genomic regions (>0.2 Mb) where a strain had more than 1.5 times the median expression value.

### Luciferase assay

HEK293 cells stably expressing a luciferase gene under the control of four consecutive NFκB consensus promoter elements (Systems Biosciences) were infected with the indicated strains of *Toxoplasma gondii* in 3 MOIs (2.5, 5, 10). At 20 hpi culture supernatants were collected and cells were lysed. Luciferase activity was measured in the lysates using Promega's Luciferase Assay System.

### Western blotting

Rabbit polyclonal antibodies to GRA15 were raised against the peptide with the amino acid sequence of GRA15_493–510_ (CGPPRTENPRQPQVPGENS) and affinity purified with the antigen (YenZym Antibody, San Francisco, CA). 2.5×10^6^ parasites were harvested from T25 flasks with infected HFFs, lysed by addition of lysis buffer, boiled for 5 minutes, placed at 4°C for 5 minutes and centrifuged at 13,000 rpm at 4°C for 15 minutes. Lysates were run on 10% SDS-PAGE protein gel, transferred to a polyvinylidene difluoride membrane and blocked in PBS/0.1% Tween-20/5% nonfat dry milk. The blot was then incubated with primary polyclonal anti-rabbit GRA15 antibodies overnight at room temperature using 1 ug/mL, followed by secondary goat anti-rabbit horseradish peroxidase antibody for 1 hour at room temperature. The blot was then incubated with a luminal based substrate (Immun-Star WesternC, Bio-Rad Laboratories) and chemiluminescence was detected using a charge-coupled device camera (Chemidoc XRS, Bio-Rad Laboratories). Bands were visualized using Quantity One 1-D analysis software.

### Immunofluorescence assays

HFFs seeded on coverslips were infected with *T. gondii* and incubated for different time points. Cells were then fixed with 4% formaldehyde in PBS for 20 min at room temperature, permeabilized and blocked with phosphate-buffered saline (PBS) plus 5% goat serum, 3% serum bovine albumin and 0.2% Triton X-100. Subsequently coverslips were incubated with primary antibody overnight at 4°C, washed twice with PBS, and incubated with fluorescent secondary antibodies and Hoechst to allow visualization of antigen and DNA respectively. Coverslips were mounted on a glass slide with Vectashield (VectorLaboratories). Photographs were taken using NIS-Elements software (Nikon) and a digital camera (CoolSNAP EZ; Roper Industries) connected to an inverted fluorescence microscope (model eclipse Ti-S; Nikon).

### Cytokine production

Cell culture supernatants were assayed for cytokines with DuoSet ELISA kits from R&D Systems according to the manufacturer's instructions. IFNβ was quantified from cell culture supernatants using a custom ELISA as described elsewhere [82].

### Quantitative PCR

Cells were infected with the indicated strains of *Toxoplasma gondii* or transfected with parasite DNA using either Xtreme-GENE9 or DOTAP (Roche) transfection reagents according to manufacturer instructions. Eight to 20 hours post infection cells were lysed, total RNA was extracted using the RNeasy Plus kit (Qiagen), and quantified using a NanoDrop spectrophotometer (Thermo Scientific). cDNA was synthetized using the SuperScript III First Strand Synthesis System (Invitrogen), and RT-PCR analysis was performed in a Light Cycler 480 II Real-time PCR machine (Roche) using the SYBR Green I Master kit (Roche) according to the manufacturer's instructions. Primers used are as follows: mIFNβ-Fw: ATAAGCAGCTCCAGCTCCAA; mIFNβ-Rv: CTGTCTGCTGGTGGAGTTCA; mRsad2-Fw: AACCCCCGTGAGTGTCAACTA; mRsad2-Rv: AACCAGCCTGTTTGAGCAGAA; mActin-Fw: TTGAACATGGCATTGTTACCAA; mActin-Rv: TGGCATAGAGGTCTTTACGGA.

### Microarrays

Human foreskin fibroblasts grown to confluency into T25 flasks were infected with the indicated strains of *T. gondii* at 3 different MOIs. At 24 h post infection total RNA was isolated using Trizol (Invitrogen) and cleaned up using RNeasy MinElute kit (Qiagen) following manufacturer's protocols. Subsequently RNA was labeled, hybridized to a human Affymetrix array (Human U133A 2.0) and processed as described elsewhere [32].

### Parasite growth

Monolayers of HFFs grown on coverslips were infected with the type II (PA7) strain or COUGAR, fixed at different time points and stained with antibodies against GRA7 and SAG1. Parasite vacuoles (GRA7 positive) per field and the number of parasites per vacuole (SAG1 staining) were counted.

### Accession numbers

The sequences reported in this paper have been deposited in Short Read Archive, http://www.ncbi.nlm.nih.gov/sra (accession Nos. SRP008923 and SRP011061)

## Supporting Information

Figure S1 ***Toxoplasma*** **induced macrophage gene expression analysis by RNAseq correlates well with microarray analysis but is more sensitive.** Venn diagram depicting number of genes that were upregulated at least 1.6 fold upon infection of BMDMs with ME49 strain when compared to non-infected cells, detected by either microarray or RNA sequencing. Pearson analysis of ranked expression values of host cell protein coding genes from samples infected with ME49 strain obtained using either microarray or RNA sequencing showed a correlation of 0.88 demonstrating that RNAseq and microarray results are comparable.
(TIF)

Figure S2 **Transcriptome analysis of host cells infected with 29** ***Toxoplasma*** **strains reveals clusters of co-regulated genes enriched in functional annotation.** Representative heat maps are shown of differentially expressed mouse gene clusters. Results of the analysis of enrichment in functional annotation using DiRE, GSEA, and Ingenuity Pathway Analysis are shown.
(TIF)

Figure S3 **Clusters of co-regulated** ***Toxoplasma*** **genes identified by transcriptome analysis.** Representative heat maps are shown of differentially expressed *Toxoplasma* gene clusters. Results of the analysis of enrichment in functional annotation using DiRE, GSEA, and Ingenuity Pathway Analysis are shown.
(TIF)

Figure S4 **Evolutionary bootstrap consensus tree of GRA15.** The tree was inferred from 500 replicates using the Neighbor-Joining method. The percentages of replicate trees in which the associated taxa clustered together in the bootstrap test (500 replicates) are shown next to the branches. The tree is drawn to scale, with branch lengths in the same units as those of the evolutionary distances used to infer the phylogenetic tree. The evolutionary distances were computed using the Poisson correction method and are in the units of the number of amino acid substitutions per site. Analyses were performed in MEGA5 software.
(TIF)

Figure S5 **Alignment of GRA15 predicted protein sequences from different** ***Toxoplasma*** **strains.** The amino acid sequences were predicted from nucleotide sequences obtained by PCR sequencing, and the multiple sequence alignment was performed using the MacVector Software (vs12.6, Accelrys, Cary, NC, USA). Identical (.) and missing (-) aminoacids are indicated.
(TIF)

Figure S6 **Alleles of GRA15 differ in their ability to induce nuclear translocation of NFκB.** Human foreskin fibroblasts were infected with the indicated strains of *Toxoplasma* for 16 hours, fixed, and stained with α-NFκB (p65, red), α-SAG1 (green) and Hoechst (blue). Nuclear translocation of p65 was only observed in cells infected with parasites expressing the type II allele of GRA15.
(TIF)

Figure S7 **Alignment of ROP16 predicted protein sequences from different** ***Toxoplasma*** **strains.** The amino acid sequences were predicted from nucleotide sequences obtained by PCR sequencing, and the multiple sequence alignment was performed using the MacVector Software (vs12.6, Accelrys, Cary, NC, USA). Identical (.) and missing (-) aminoacids are indicated.
(TIF)

Figure S8 **Sustained nuclear translocation of STAT6 can be achieved by either type I or atypical alleles of ROP16, but not by the type II allele.** Human foreskin fibroblasts were infected with the indicated strains of *Toxoplasma* for 16 hours, fixed, and stained with α-SAG1 (green), Hoechst (blue) and either α-phosphorylated STAT6 (red). Canonical types I and III parasites and all atypical strains tested, but not type II parasites, induced sustained nulcear translocation of STAT6. Type I parasites knockout out for ROP16_I_ loose their ability to activate STAT6, whereas Type II parasites overexpressing ROP16_I_ acquire the ability to do so.
(TIF)

Figure S9 **Sustained nuclear translocation of STAT5 can be achieved by either type I or atypical alleles of ROP16, but not by the type II allele.** Human foreskin fibroblasts were infected with the indicated strains of *Toxoplasma* for 16 hours, fixed, and stained with α-SAG1 (green), Hoechst (blue) and either α-phosphorylated STAT5 (red). Canonical types I and III parasites and all atypical strains tested, but not type II parasites, induced sustained nulcear translocation of STAT5. Type I parasites knockout out for ROP16_I_ loose their ability to activate STAT5, whereas Type II parasites overexpressing ROP16_I_ acquire the ability to do so.
(TIF)

Figure S10 **Type I interferon prodution induced by double stranded DNA is dependent on RIG-I.** Synthetic double stranded DNA poly d(A):d(T) was transfected into immortalized macrophages either wild type or knockout for the indicated genes using XtremeGENE9. Cells were incubated overnight, subsequently total RNA was extracted and qPCR was performed to detect *Ifnb1*. Relative expression levels were calculated by normalizing against actin expression.
(TIF)

Figure S11 **RNA profile of** ***Toxoplasma*** **-infected bone-marrow derived macrophages.** Bone marrow-derived macrophages either non-infected or infected with three different MOIs for 20 h with the indicated strains were lysed, and cells extracts subjected to total RNA isolation using Qiagen RNeasy Plus kit. Integrity, sizing, quality and quantification of RNA was then performed using the Agilent 2100 Bioanalyser, and examples of the pseudo gel image created for a non-infected (NI) or infected (GUYDOS, VAND and COUGAR) samples are shown. Only samples with equivalent amounts of parasite-derived RNA were used for RNA sequencing.
(TIF)

Protocol S1 **Detailed protocol used for RNA sequencing and expression profiling of** ***Toxoplasma gondii*** **and infected host cells.**
(DOC)

Table S1 **Expression values for all** ***Toxoplasma*** **and mouse genes.** FPKM values for *Toxoplasma* and mouse genes for each of the samples infected with one of the 29 different *Toxoplasma* strains are indicated. Also the position of each gene in each of the co-regulated *Toxoplasma* or mouse gene expression clusters is indicated. Potential *Toxoplasma* or host gene expression regulators are also indicated.
(TXT)

Table S2 **Pair-wise Pearson correlations between average expression values of the mouse and** ***Toxoplasma*** **gene expression clusters.**
(XLS)

Table S3 **Parameters used running Tophat and Cufflinks to obtain FPKM values for (A) mouse or (B)** ***Toxoplasma*** **genes.**
(XLS)
