# Supplementary figures and images for: Transcriptional Analysis of Murine Macrophages Infected with Different Toxoplasma Strains Identifies Novel Regulation of Host Signaling Pathways
Source: PLoS Pathog. 2013 Dec 19;9(12):e1003779. doi: 10.1371/journal.ppat.1003779 (PMC3868521; doi:10.1371/journal.ppat.1003779)

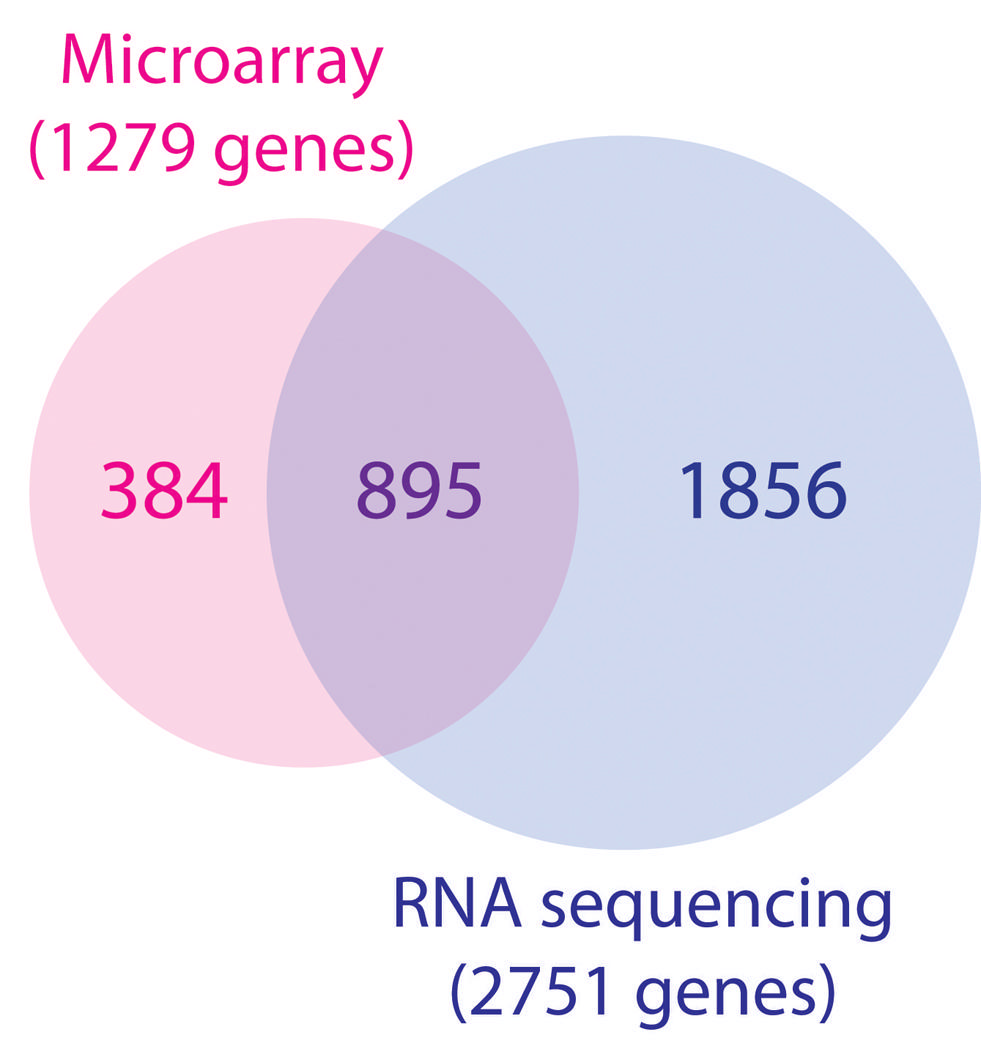

Supplement: Figure S1 — Toxoplasma induced macrophage gene expression analysis by RNAseq correlates well with microarray analysis but is more sensitive. Venn diagram depicting number of genes that were upregulated at least 1.6 fold upon infection of BMDMs with ME49 strain when compared to non-infected cells, detected by either microarray or RNA sequencing. Pearson analysis of ranked expression values of host cell protein coding genes from samples infected with ME49 strain obtained using either microarray or RNA sequencing showed a correlation of 0.88 demonstrating that RNAseq and microarray results are comparable. (TIF) [file ppat.1003779.s001.tif]

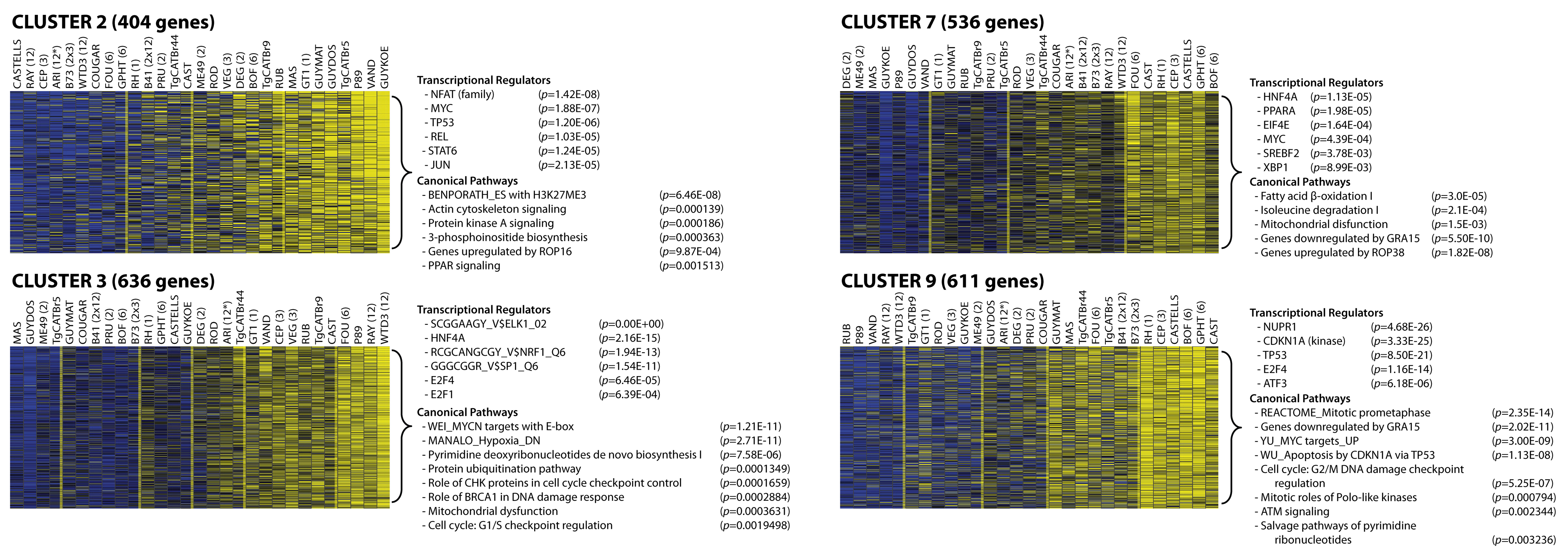

Supplement: Figure S2 — Transcriptome analysis of host cells infected with 29 Toxoplasma strains reveals clusters of co-regulated genes enriched in functional annotation. Representative heat maps are shown of differentially expressed mouse gene clusters. Results of the analysis of enrichment in functional annotation using DiRE, GSEA, and Ingenuity Pathway Analysis are shown. (TIF) [file ppat.1003779.s002.tif]

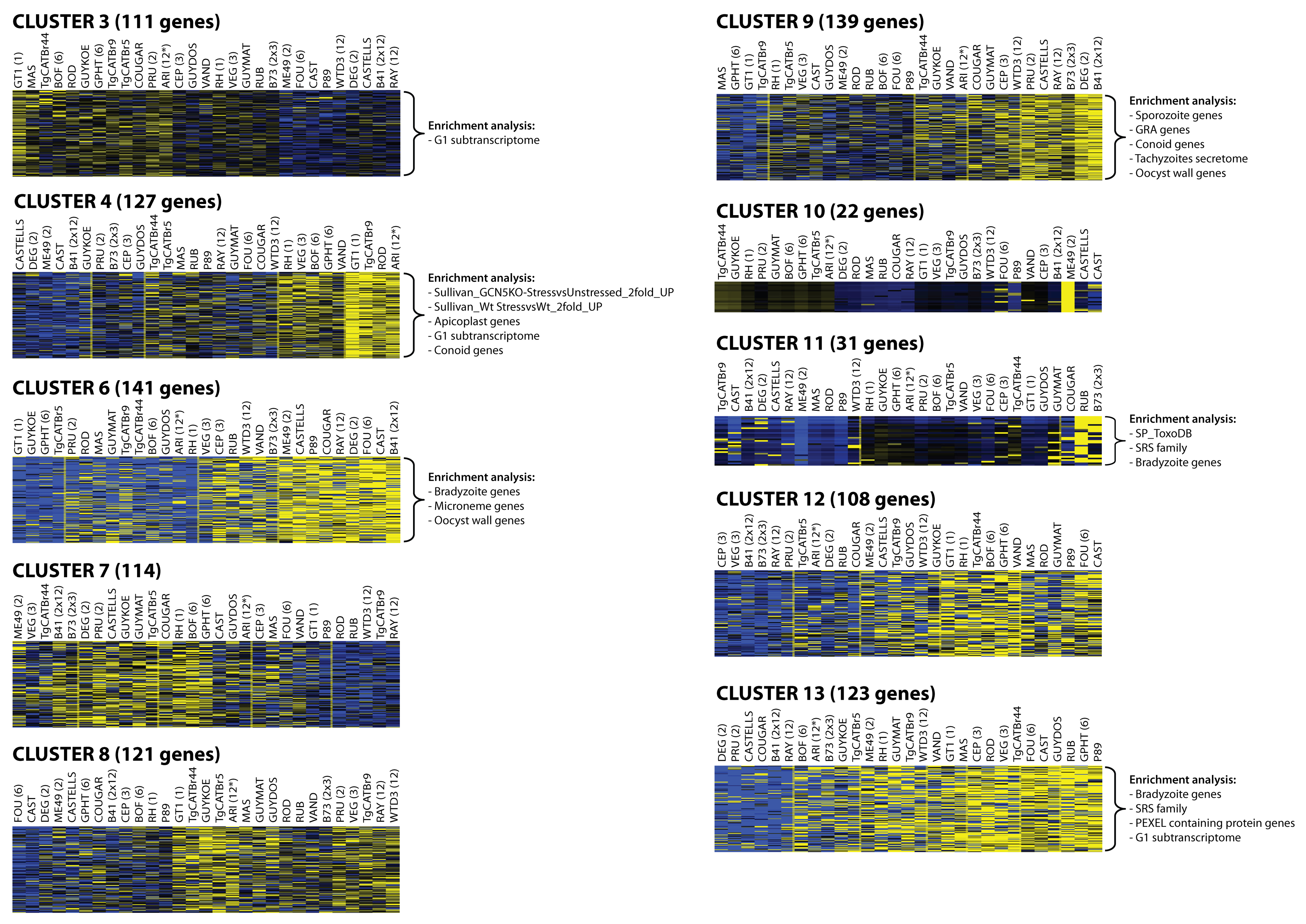

Supplement: Figure S3 — Clusters of co-regulated Toxoplasma genes identified by transcriptome analysis. Representative heat maps are shown of differentially expressed Toxoplasma gene clusters. Results of the analysis of enrichment in functional annotation using DiRE, GSEA, and Ingenuity Pathway Analysis are shown. (TIF) [file ppat.1003779.s003.tif]

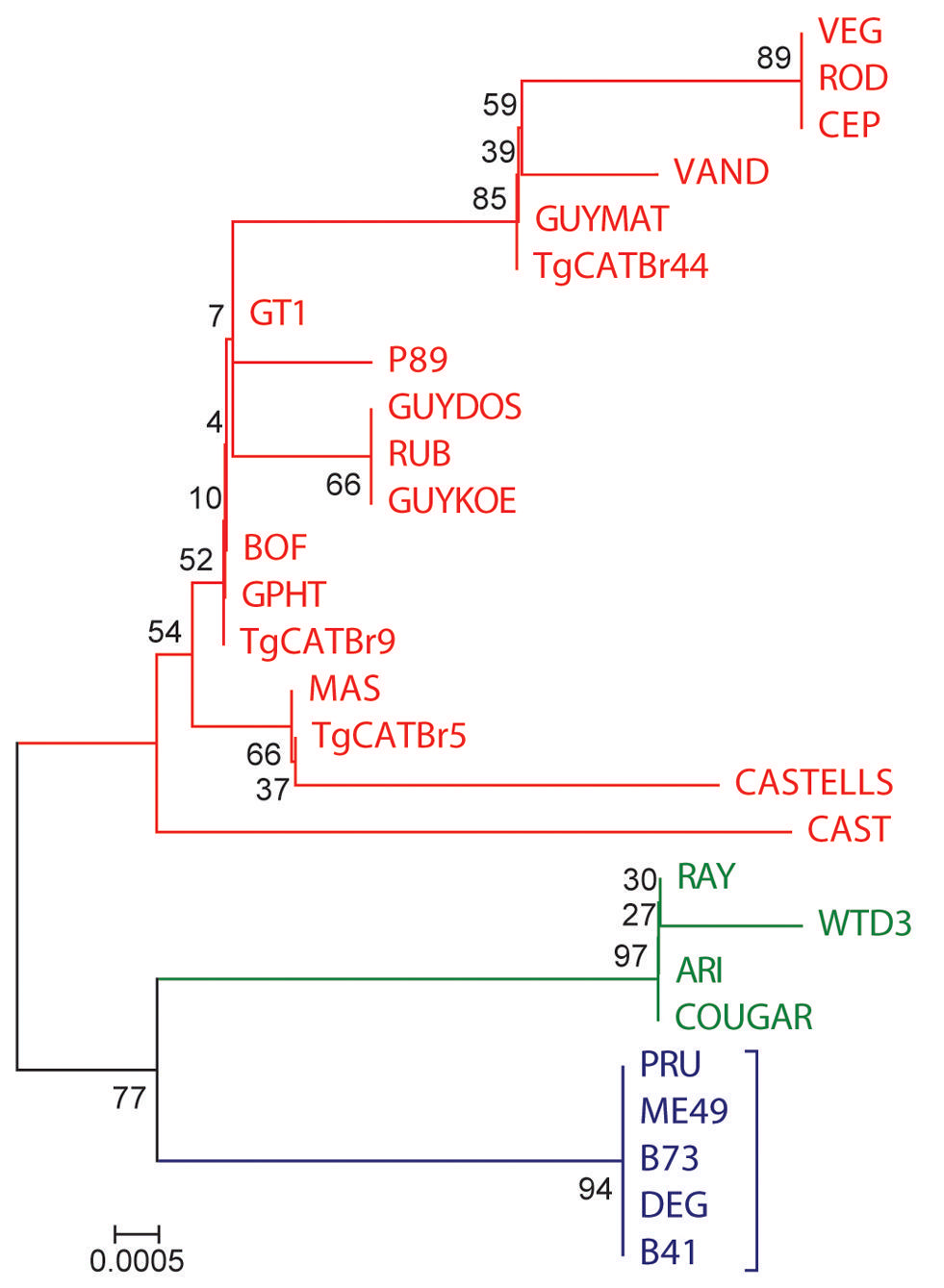

Supplement: Figure S4 — Evolutionary bootstrap consensus tree of GRA15. The tree was inferred from 500 replicates using the Neighbor-Joining method. The percentages of replicate trees in which the associated taxa clustered together in the bootstrap test (500 replicates) are shown next to the branches. The tree is drawn to scale, with branch lengths in the same units as those of the evolutionary distances used to infer the phylogenetic tree. The evolutionary distances were computed using the Poisson correction method and are in the units of the number of amino acid substitutions per site. Analyses were performed in MEGA5 software. (TIF) [file ppat.1003779.s004.tif]

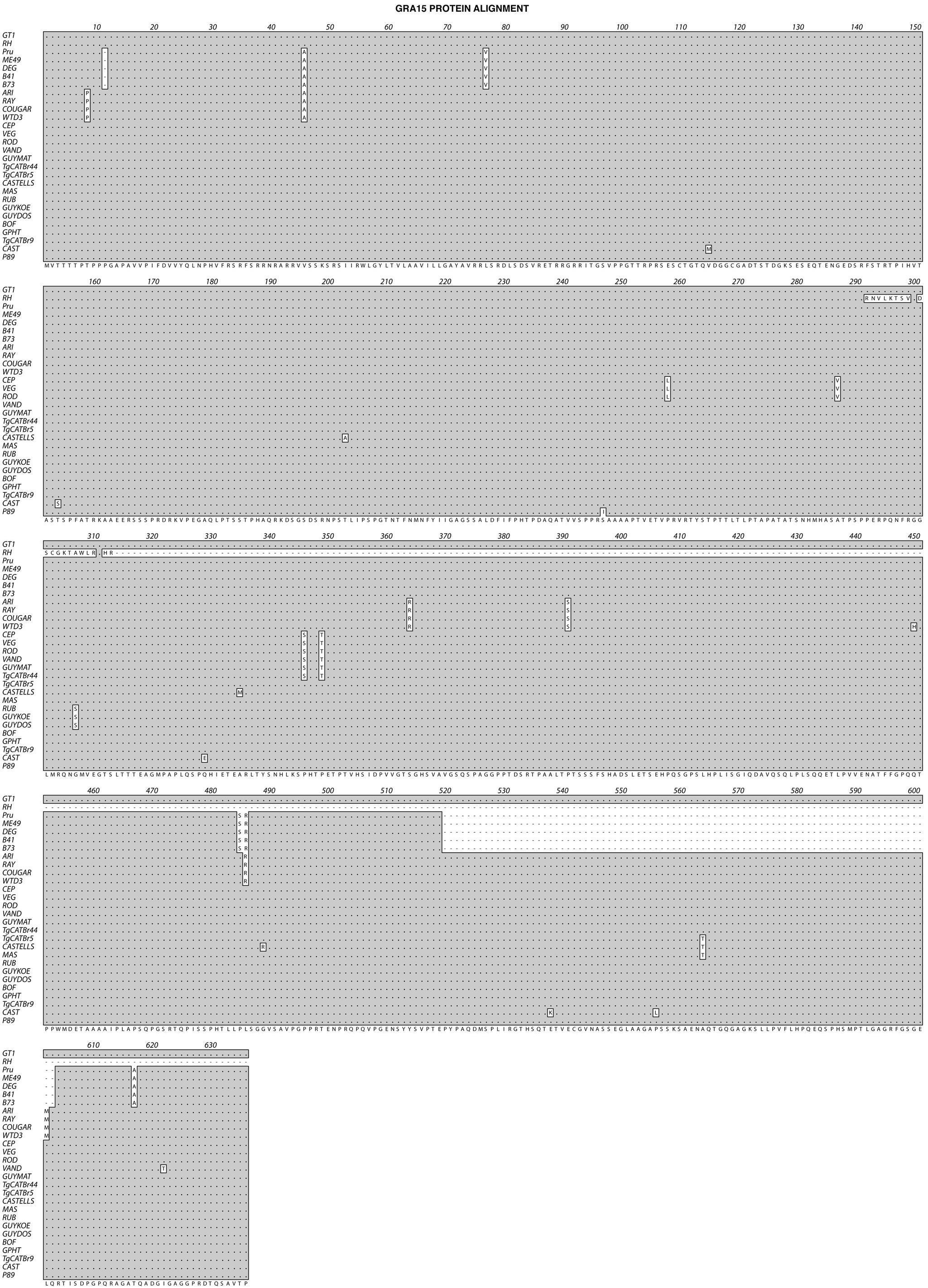

Supplement: Figure S5 — Alignment of GRA15 predicted protein sequences from different Toxoplasma strains. The amino acid sequences were predicted from nucleotide sequences obtained by PCR sequencing, and the multiple sequence alignment was performed using the MacVector Software (vs12.6, Accelrys, Cary, NC, USA). Identical (.) and missing (-) aminoacids are indicated. (TIF) [file ppat.1003779.s005.tif]

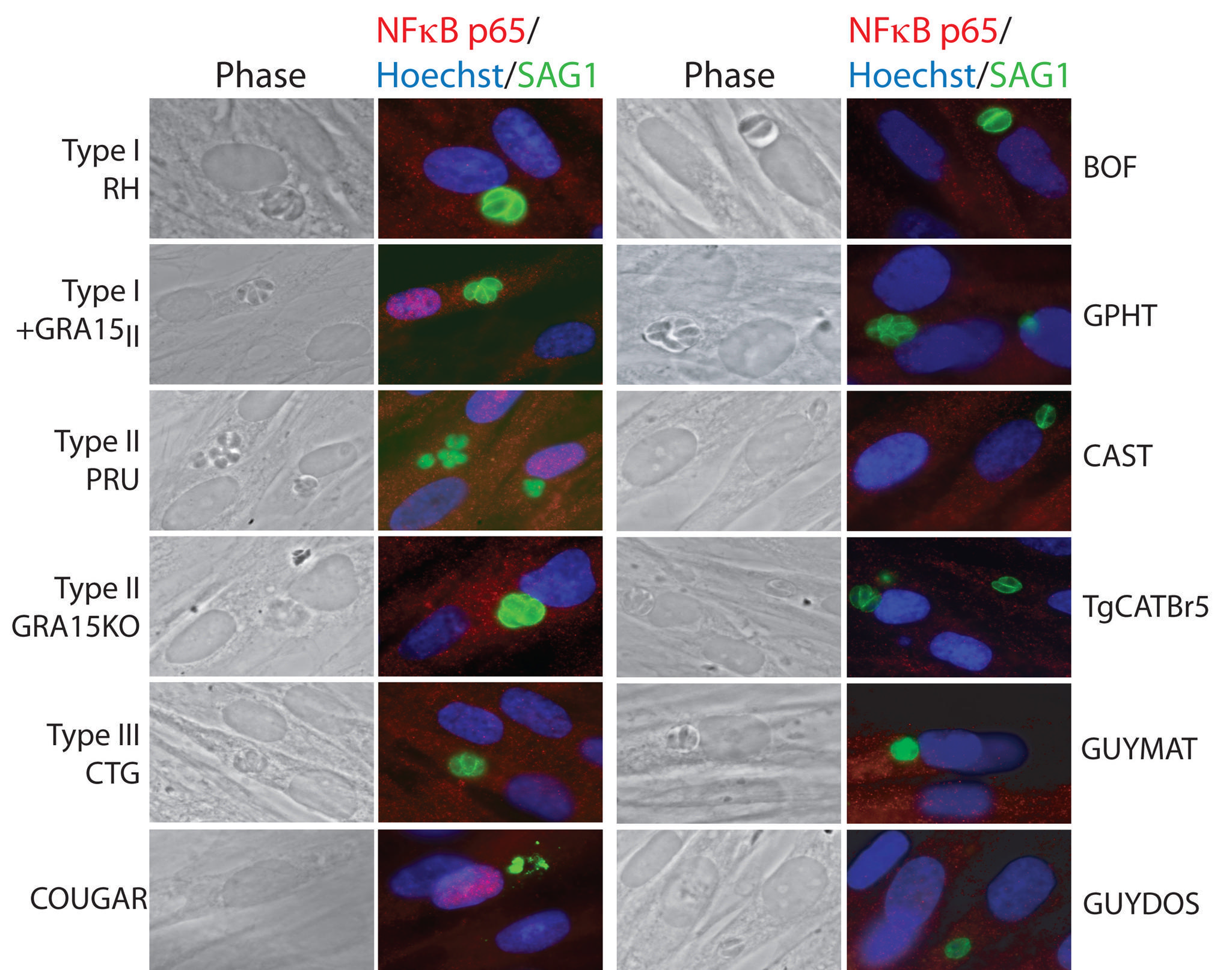

Supplement: Figure S6 — Alleles of GRA15 differ in their ability to induce nuclear translocation of NFκB. Human foreskin fibroblasts were infected with the indicated strains of Toxoplasma for 16 hours, fixed, and stained with α-NFκB (p65, red), α-SAG1 (green) and Hoechst (blue). Nuclear translocation of p65 was only observed in cells infected with parasites expressing the type II allele of GRA15. (TIF) [file ppat.1003779.s006.tif]

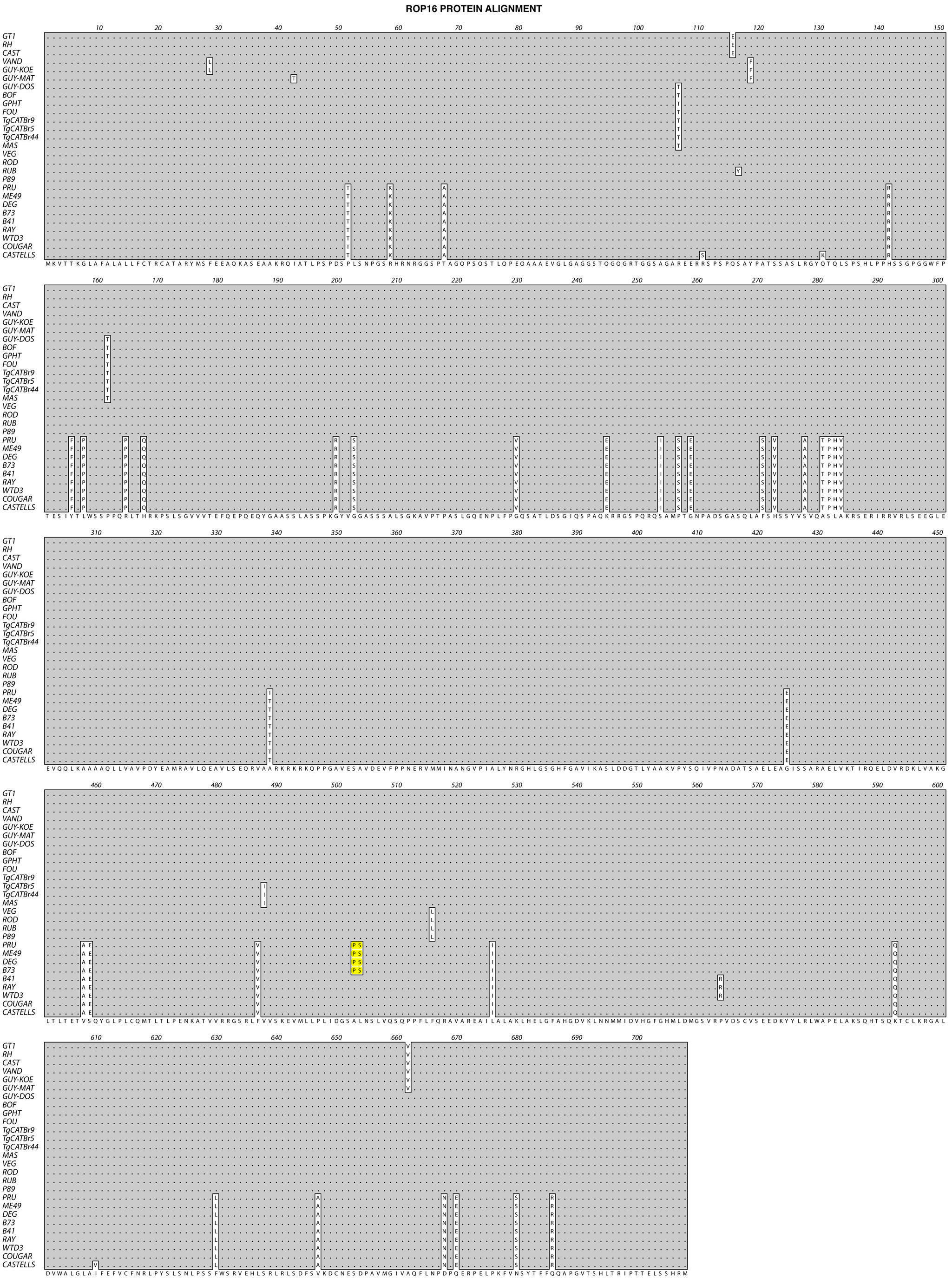

Supplement: Figure S7 — Alignment of ROP16 predicted protein sequences from different Toxoplasma strains. The amino acid sequences were predicted from nucleotide sequences obtained by PCR sequencing, and the multiple sequence alignment was performed using the MacVector Software (vs12.6, Accelrys, Cary, NC, USA). Identical (.) and missing (-) aminoacids are indicated. (TIF) [file ppat.1003779.s007.tif]

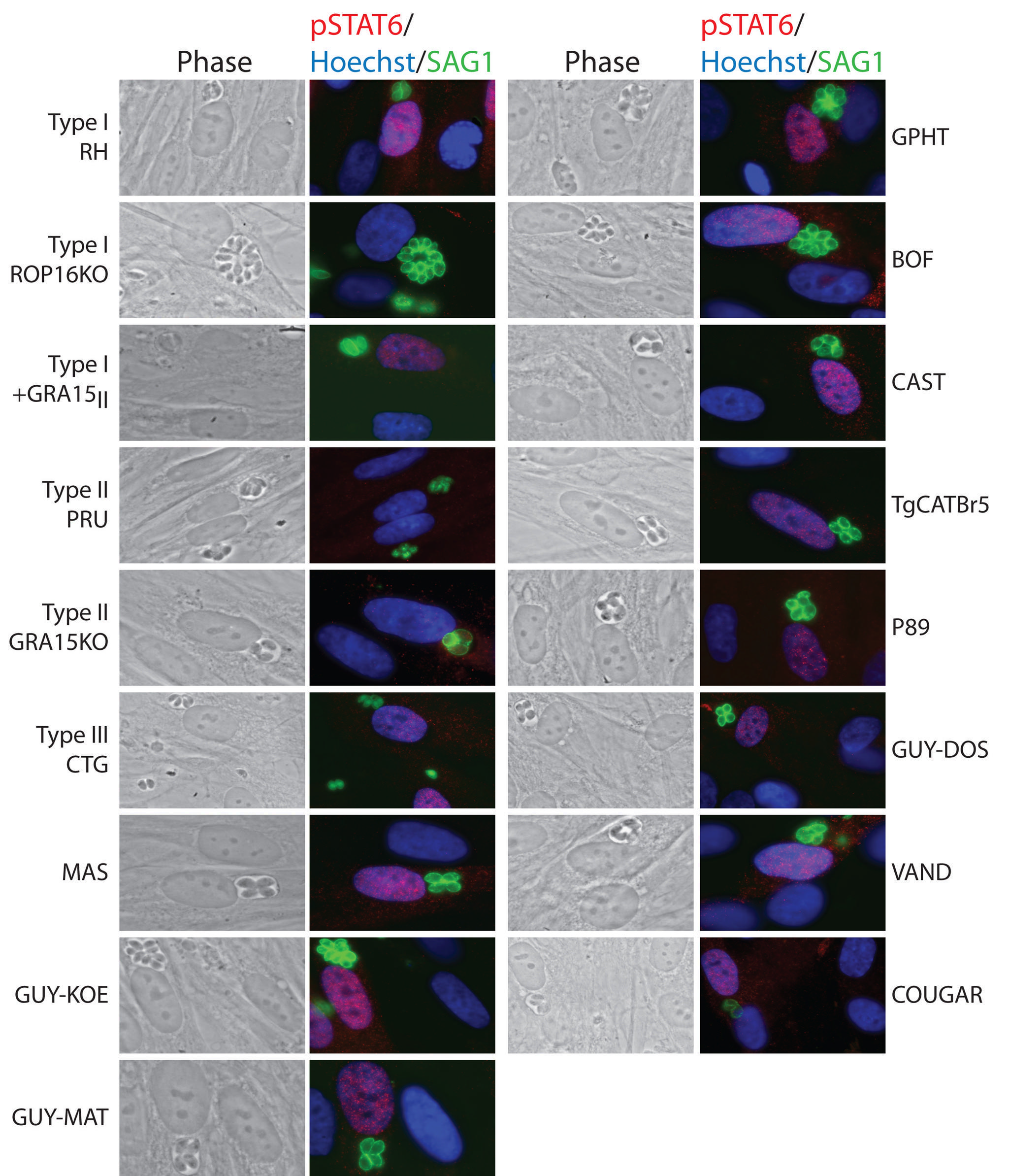

Supplement: Figure S8 — Sustained nuclear translocation of STAT6 can be achieved by either type I or atypical alleles of ROP16, but not by the type II allele. Human foreskin fibroblasts were infected with the indicated strains of Toxoplasma for 16 hours, fixed, and stained with α-SAG1 (green), Hoechst (blue) and either α-phosphorylated STAT6 (red). Canonical types I and III parasites and all atypical strains tested, but not type II parasites, induced sustained nulcear translocation of STAT6. Type I parasites knockout out for ROP16I loose their ability to activate STAT6, whereas Type II parasites overexpressing ROP16I acquire the ability to do so. (TIF) [file ppat.1003779.s008.tif]

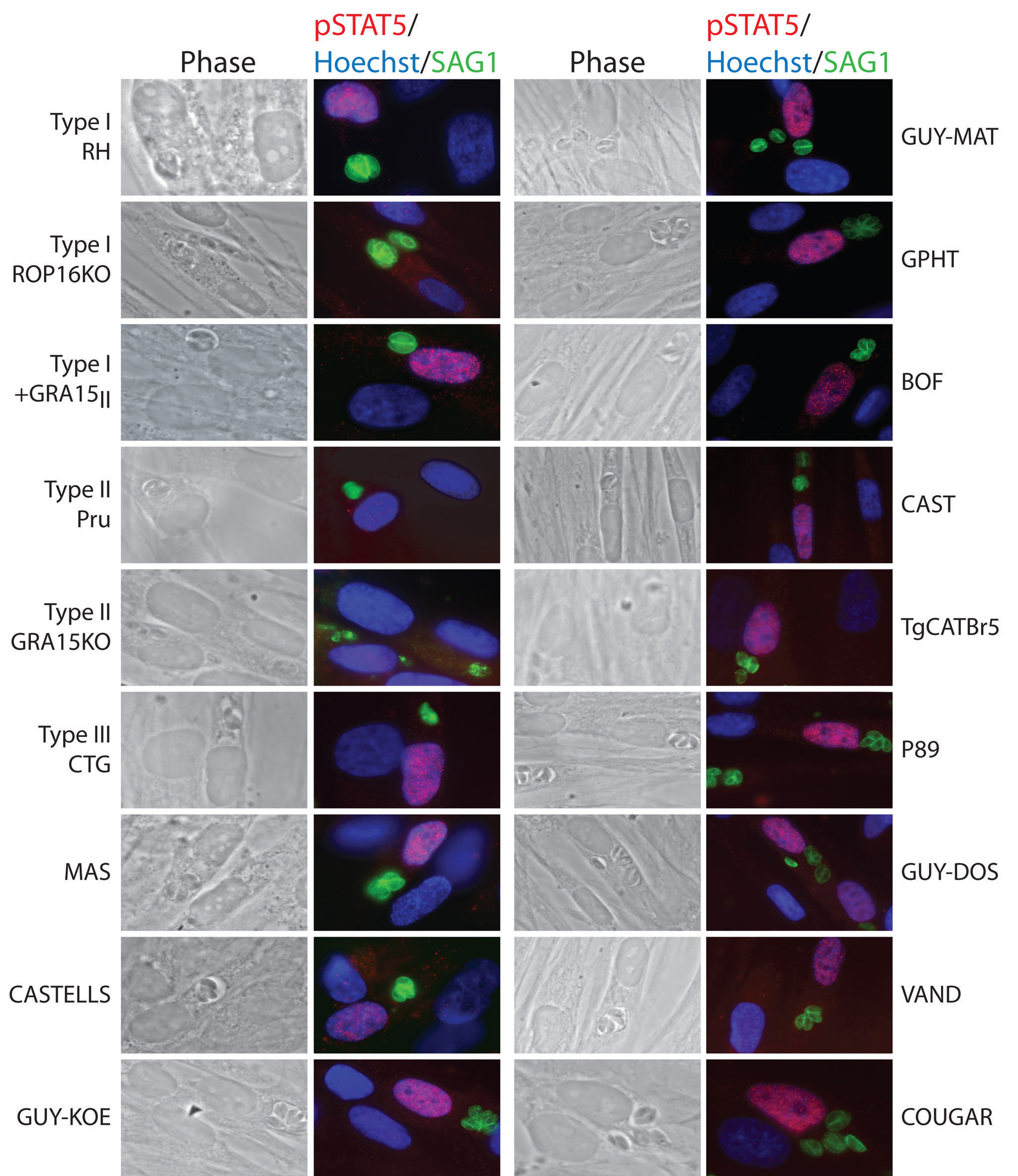

Supplement: Figure S9 — Sustained nuclear translocation of STAT5 can be achieved by either type I or atypical alleles of ROP16, but not by the type II allele. Human foreskin fibroblasts were infected with the indicated strains of Toxoplasma for 16 hours, fixed, and stained with α-SAG1 (green), Hoechst (blue) and either α-phosphorylated STAT5 (red). Canonical types I and III parasites and all atypical strains tested, but not type II parasites, induced sustained nulcear translocation of STAT5. Type I parasites knockout out for ROP16I loose their ability to activate STAT5, whereas Type II parasites overexpressing ROP16I acquire the ability to do so. (TIF) [file ppat.1003779.s009.tif]

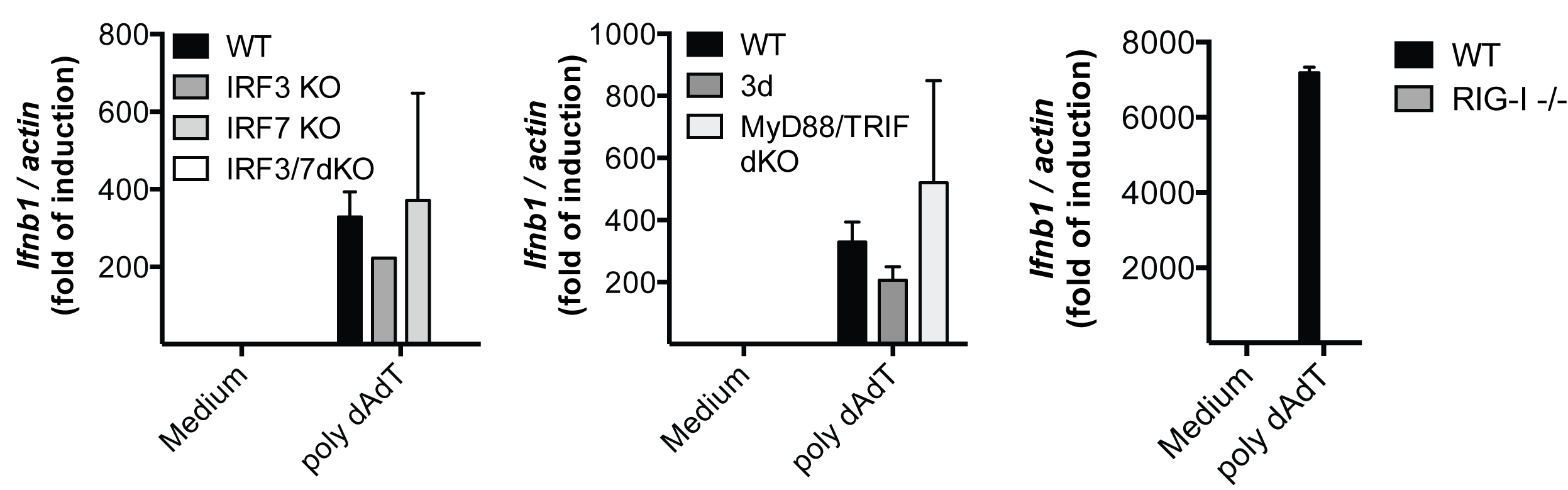

Supplement: Figure S10 — Type I interferon prodution induced by double stranded DNA is dependent on RIG-I. Synthetic double stranded DNA poly d(A):d(T) was transfected into immortalized macrophages either wild type or knockout for the indicated genes using XtremeGENE9. Cells were incubated overnight, subsequently total RNA was extracted and qPCR was performed to detect Ifnb1. Relative expression levels were calculated by normalizing against actin expression. (TIF) [file ppat.1003779.s010.tif]

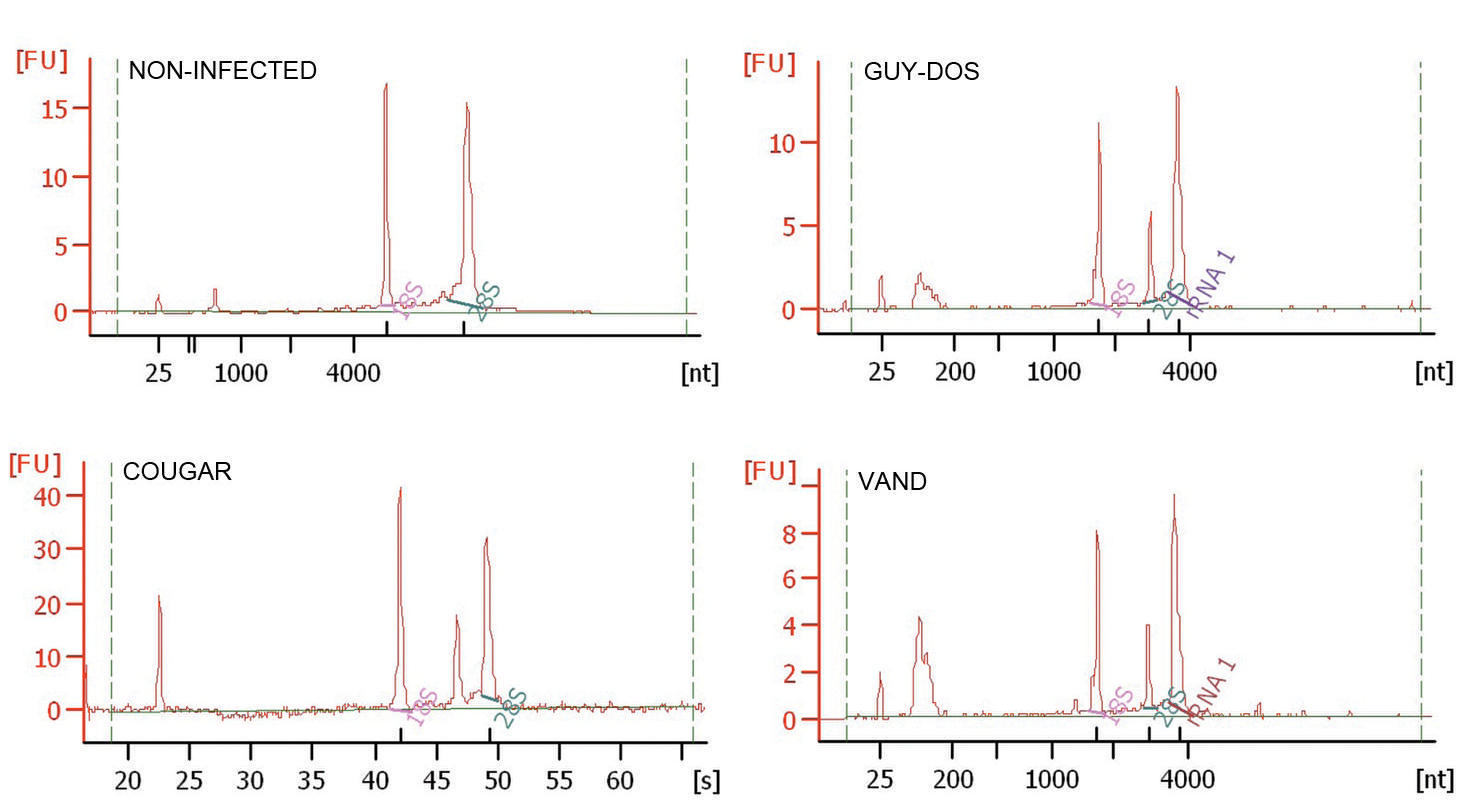

Supplement: Figure S11 — RNA profile of Toxoplasma -infected bone-marrow derived macrophages. Bone marrow-derived macrophages either non-infected or infected with three different MOIs for 20 h with the indicated strains were lysed, and cells extracts subjected to total RNA isolation using Qiagen RNeasy Plus kit. Integrity, sizing, quality and quantification of RNA was then performed using the Agilent 2100 Bioanalyser, and examples of the pseudo gel image created for a non-infected (NI) or infected (GUYDOS, VAND and COUGAR) samples are shown. Only samples with equivalent amounts of parasite-derived RNA were used for RNA sequencing. (TIF) [file ppat.1003779.s011.tif]
