# Supplementary material for: Transcriptional Analysis of Murine Macrophages Infected with Different Toxoplasma Strains Identifies Novel Regulation of Host Signaling Pathways
Source: PLoS Pathog. 2013 Dec 19;9(12):e1003779. doi: 10.1371/journal.ppat.1003779 (PMC3868521; doi:10.1371/journal.ppat.1003779)
Supplement: Protocol S1 — Detailed protocol used for RNA sequencing and expression profiling of Toxoplasma gondii and infected host cells. (DOC) [file ppat.1003779.s012.doc]

**Protocol S1**

**High-throughput RNA sequencing.** Cells were infected with *Toxoplasma* using three different MOIs (15, 10 and 7.5). RNA quality and concentration was verified using the Agilent 2100 Bioanalyzer. Note that the Bioanalyzer results can clearly distinguish the *Toxoplasma* ribosomal large subunit from the host large ribosomal subunit, which can then be used to estimate the amount of *T. gondii* RNA versus host RNA. Based on the plaque results and the Bioanalyzer results for each strain one out of the 3 MOIs samples was chosen, for RNA-sequencing, so all the samples had equivalent infection rates (Figure S11). After processing, RNA samples were barcoded and multiplexed in a single lane on an Illumina sequencing flow cell for paired end sequencing on an Illumina High-seq instrument. In our experiments using 4 samples per lane we got enough (~220 million paired-end reads/lane) read density for reliable gene expression analysis. The Illumina sequencing pipeline performed primary data acquisition, determined base calls and calculated confidence scores. In infected samples, ~69% of the reads aligned to the mouse genome, whereas ~18% of the reads aligned to the parasite genome. Since parasites are grown in monolayers of human fibroblasts, we also aligned the reads against the human genome and we observed ~5% contamination with human RNA.

**Expression profiling of *Toxoplasma gondii* strains and infected host cells.** To identify host cell signaling pathways modulated by *T. gondii* in a strain specific manner, we infected primary bone marrow-derived macrophages (BMDMs) with 29 different *T. gondii* strains, representing global diversity, and determined the macrophage and *Toxoplasma* transcriptome 20hs post infection using high-throughput RNA sequencing. Besides the advantage of being able to detect SNPs, splice isoforms and to profile both host and parasite transcriptome at the same time, comparing our RNA-seq data to our previously published Affymetrix mouse and *T. gondii* arrays data showed that both the sensitivity and dynamic range of our RNA-seq were significantly better, as has been described by others . For instance, using Affymetrix mouse or *T. gondii* arrays we are able to detect ~ 9,000 or 4,500 genes above background, respectively, whereas using RNA-seq we detected more than 12,000 mouse genes and approximately 8,000 *T. gondii* genes.

Aligning reads from different *Toxoplasma* strains to the type II ME49 reference genome creates bias. Since our alignment settings allow 2 mis-matches per read, when the RNA-seq data of strains that are highly divergent are aligned to the ME49 genome many reads might have more than 2 mis-matches, therefore reducing the FPKM values for that strain. To investigate the effect of this we generated a synthetic *Toxoplasma* genome, in which each SNP was substituted by an alternative base (different from both ME49 and the SNP). We then aligned our dataset to either the ME49 or the synthetic genome and determined FPKM values. Background level was arbitrarily determined to be 1.8 FPKM. Non protein-coding genes and genes with maximum expression level across all strains below background were excluded from the analysis, resulting in a final database with expression values for 7814 genes. Correlation analysis of expression values obtained after alignment of the reads against ME49 (version 8.0) versus our synthetic genome showed that expression values for only a few genes (456), mainly genes coding for small and/or very polymorphic proteins, were significantly affected by the genome chosen for read alignment. Given that more than 94% of the genes (7358) displayed a correlation of 0.7 or above, we performed all subsequent analysis with the data obtained using synthetic genome as the reference.

Alignment of reads to the mouse genome (build 37.2) gave back expression values for 25791 genes. From this dataset we excluded all non protein-coding genes and genes with maximum expression level across all strains below 1.8. We observed that both the level of residual human cell contamination and experimental batch accounted for a significant level of variation in our data. Therefore, to avoid any bias in our dataset we first performed a correlation analysis of expression values against percentage of human contamination in each sample, and excluded all genes with correlation above 0.7 (557 genes). Subsequently we performed an analysis of significance using Multiple Experiment Viewer (MeV) to identify genes strongly correlated with experimental batch. 859 genes that strongly correlated with batch were also excluded, leaving a final dataset with expression values for 9644 genes.

To identify strain-specific signaling pathways modulated by *Toxoplasma* we specifically focused on macrophage genes that were at least two fold differentially regulated between two strains and had at least an FPKM of 10 in one strain. Gene expression values were changed so the minimum value for each gene for each strain was at least 1. The data was subsequently loaded in MeV, log2 transformed, column normalized and mean centered. This normalized data was subsequently loaded in Genomica, which was used to partition the data in co-regulated clusters, using regulators (known host transcriptional modulators, Table S1) to subdivide the clusters into modules where different strains had the same regulation of the genes in the cluster. These genes were then investigated for enrichment in functional annotation, such as enrichment in transcription factor binding sites (TFBS) in their promoters or belonging to a particular pathway using DiRE , GSEA , MeV and Ingenuity Pathway Analysis ([www.ingenuity.com](http://www.ingenuity.com/)). We also made an in-house database of data from microarray experiments of GRA15 , ROP16 , and ROP38-regulated genes . Parasite gene clusters were made in a similar way but we focused on parasite genes with an expression value of at least FPKM=10 in at least one strain that were at least 4-fold differentially regulated between 2 strains. We used the AP2 factors and putative regulators consisting of DNA and/or RNA-binding domain containing proteins or histone modulators as putative regulators for the *Toxoplasma* gene clusters. We also made an in-house *Toxoplasma* database consisting of genes that are up or downregulated, as determined by microarray experiments, under different conditions (*e.g.* bradyzoite induction).
